# Association between *Helicobacter pylori* antibodies and otolaryngological diseases

**DOI:** 10.1016/j.bjorl.2024.101488

**Published:** 2024-08-08

**Authors:** Fang Zhang, Haowei Zhang, Jing Luo, Yixi Xiao, Hai Liu, Jianhui Zhang

**Affiliations:** a The Third People’s Hospital of Chengdu, The Affiliated Hospital of Southwest Jiaotong University, Department of Otolaryngology Head and Neck Surgery, Chengdu, Sichuan, China; b The Affiliated Hospital of North Sichuan Medical College, Department of Otolaryngology Head and Neck Surgery, Nanchong, Sichuan, China; c China West Normal University, Nanchong, Sichuan, China

**Keywords:** Otolaryngological diseases, *Helicobacter pylori*, Mendelian randomization, Causal relationship, Risk factors

## Abstract

**Objective:**

Observational studies have shown that *Helicobacter pylori* is related to some otolaryngological diseases. However, it is unclear if *H. pylori* infection causally affects these diseases. To elucidate *H. pylori* role in 12 common otolaryngological diseases, we conducted two-sample Mendelian randomization analysis.

**Methods:**

Single-nucleotide polymorphisms associated with 7 *H. pylori* antibodies (IgG, CagA, Catalase, GroEL, OMP, UREA and Vac A) served as instrumental variables. We primarily employed random-effects inverse variance weighting for causal estimation, supplemented by MR Egger, weighted median, simple mode, and weighted mode. Sensitivity analyses, including heterogeneity, pleiotropy, and leave-one-out tests, validated robustness.

**Results:**

MR analysis using inverse variance weighting (random effects) revealed genetically predicted *H. pylori* CagA antibodies correlated with increased risk of nonsuppurative otitis media (OR = 1.0778, 95% CI 1.0114–1.1487, *p*-value = 0.021). No causal relationship was observed between *H. pylori* antibodies and other common otolaryngological diseases. Sensitivity analyses found no pleiotropy or heterogeneity, affirming result reliability.

**Conclusion:**

This study suggests that the levels of *H. pylori* CagA antibodies may contribute to the development of nonsuppurative otitis media. Further studies are needed in the future to elucidate the specific mechanism of *H. pylori* in this disease.

**Level of evidence:**

Level III.

## Highlights

• This study reveals a causal link between *H. pylori* CagA antibodies and nonsuppurative otitis media.
• No causal relationship between *H. pylori* antibodies and 11 other common otolaryngological diseases.
• Sensitivity analyses confirm the robustness and reliability of the findings.

## Introduction

*Helicobacter pylori* (*H. pylori*) is a Gram-negative spiral-shaped bacterium widely recognized as one of the major pathogenic factors for gastrointestinal diseases such as gastritis, peptic ulcers, and gastric cancer.^1^, ^2^ Since its discovery in human gastric mucosa by Marshall and Warren in 1982,^3^ research on its pathogenic mechanisms and clinical significance has deepened. However, in addition to its impact on the gastrointestinal system, an increasing number of studies in recent years suggest that *H. pylori* may also be closely related to diseases of other organ systems in the human body, such as neurological diseases,^4^, ^5^ dermatological diseases,^6^ cardiovascular diseases,^7^ and metabolic diseases.^8^

Previous studies have found that *H. pylori* also exists in the upper respiratory system.^9^ The relationship between *H. pylori* and some otolaryngological diseases has also been explored, but the results are conflicting. For instance, a case-control study suggested that *H. pylori* is one of the important bacteria affecting the pathogenesis of Otitis Media with Effusion (OME),^10^ while another study indicated a possible association between *H. pylori* infection and chronic sinusitis with nasal polyps.^11^ However, other studies seem to contradict the role of *H. pylori* in OME^12^ and sinusitis.^13^ These data presented are all sourced from observational studies, which are susceptible to biases due to confounding factors and reverse causality, potentially leading to inaccurate causal inferences.^14^ Furthermore, there are many common risk factors for otolaryngological conditions, such as smoking and alcohol consumption,^15^ which increase the likelihood of residual confounding. Moreover, if *H. pylori* infection occurs subsequent to the onset of the disease, reverse causality may be present. While Randomized Controlled Trials (RCTs) are highly regarded as the gold standard in medical research, they are limited due to high economic costs, resource intensiveness, and lengthy durations. Additionally, ethical considerations pose a significant barrier, rendering intentional *H. pylori* infection of populations impractical. Therefore, when RCTs are constrained by methodological or ethical limitations, genetic data studies serve as an important analytical method to strengthen causal inference in observational research.^16^

Mendelian Randomization (MR) is a genetic epidemiological study method that uses genetic variants as Instrumental Variables (IVs) to explore the causal relationship between exposure factors and disease outcomes.^17^ Because alleles for genetic variants associated with exposure factors are randomly assigned at the time of conception, they are less susceptible to confounding factors and conform to the normal causal order.^18^, ^19^ With the increasing availability of aggregated data from large consortia,^20^ Genome-Wide Association Studies (GWASs) can provide powerful and reliable IVs for MR research. Therefore, we conducted a two-sample MR analysis using genetic variants associated with antibodies to *H. pylori* obtained from published GWASs to assess the relationship between *H. pylori* infection and otolaryngological diseases.

## Methods

We used the public GWASs summary statistics (https://gwas.mrcieu.ac.uk/), which did not include identifiable data. Therefore, there was no need for a new ethical review, nor was it necessary to obtain informed consent.

**Figure 1:**
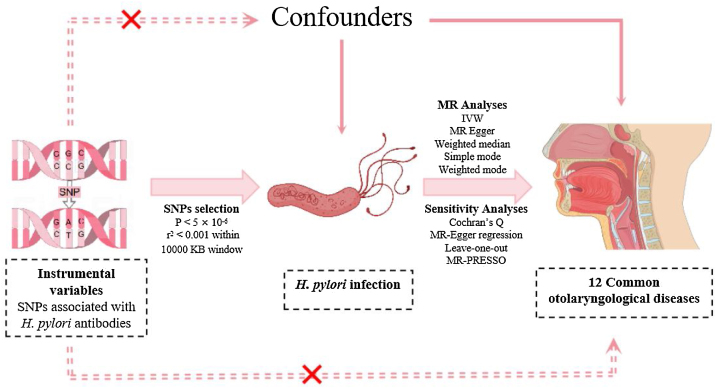
The schematic representations of this study.

MR is based on three hypotheses.^21^ Single-Nucleotide Polymorphisms (SNPs) that were associated with exposure were chosen as IVs using strict inclusion and exclusion criteria. Subsequently, several sensitivity analyses were conducted to assess result robustness. The flowchart of the study design is shown in Fig. 1.

### Exposure

Confirmation of *H. pylori* infection involves measuring serum-specific antibodies targeting *H. pylori* proteins, encompassing seven antibodies: IgG, Cag A, Catalase, GroEL, OMP, UREA and Vac A. Detailed information regarding these data can be found in Supplementary Table 1. As genetic instruments for exposure, we obtained SNPs that were associated with 7 antibody phenotypes (*p* < 5 × 10^−6^) and were independent (*r*^2^ < 0.001 within a 10,000 KB window).

### Outcomes

12 common otolaryngological diseases were identified as outcomes, including acute upper respiratory tract infection, chronic diseases of tonsils and adenoids, chronic laryngitis and laryngotracheitis, acute sinusitis, chronic sinusitis, allergic rhinitis, nasal polyps, acute suppurative otitis media, chronic suppurative otitis media, nonsuppurative otitis media, sleep apnoea and laryngeal cancer. The summary-level data for acute upper respiratory tract infection, chronic diseases of tonsils and adenoids, chronic laryngitis and laryngotracheitis, acute sinusitis, chronic sinusitis, nasal polyps, acute suppurative otitis media and nonsuppurative otitis media were obtained from the FinnGen consortium. The GWAS summary statistics for chronic suppurative otitis media involved 1108 cases and 483,037 controls.^22^ Data summaries for laryngeal cancer were sourced from the UK Biobank, and sleep apnoea and allergic rhinitis data were from the MRC-IEU Consortium. Supplementary Table 1 shows an overview of the demographics included in this study.

### MR analyses

In this study, the random-effects model Inverse-Variance Weighting (IVW) analysis^23^ was used as the primary method, supplemented by alternative methods including MR Egger,^24^ weighted median,^25^ simple mode,^26^ and weighted mode^25^ to analyze the effect of exposure on outcomes. The IVW method has been observed to be marginally more efficient than the others under specific condition.^25^ Consequently, the findings primarily relied on the IVW method, with supplementary support from other methodologies. The strength of the connection between the genetic instruments and exposure phenotypes (F statistics) was estimated to evaluate the possibility of weak instrument bias.^27^ Moreover, we used MR pleiotropy residual sum and outliers (MR-PRESSO) to discover outlier variables and to evaluate causal estimates after removing outliers.^28^

### Sensitivity analyses

For robust findings, several sensitivity analyses were conducted, including assessments of heterogeneity, horizontal pleiotropy, and the leave-one-out test. Heterogeneity was evaluated using IVW and MR Egger regression, with Cochran Q statistic quantifying heterogeneity and significance set at *p* < 0.05. The MR Egger regression's intercept term tested horizontal pleiotropy,^24^ with values near 0 (<0.1) and *p* > 0.05 indicating its absence. MR-PRESSO test was used to detect horizontal pleiotropic outliers,^28^ with 2000 distributions set. Moreover, asymmetry in funnel plots^26^ and forest plots may be a sign of horizontal pleiotropy in IVs. Leave-one-out analysis was performed to determine whether the observed correlation was modified by any individual SNP.

Odds Ratios (ORs) and their 95% Confidence Intervals (95% CIs) were used to evaluate the association between *H. pylori* and otolaryngological diseases. All MR analyses were carried out in RStudio (version 4.3.1) with R packages including TwoSampleMR (version 0.5.7) and MR-PRESSO (version 1.0).

## Results

Exposure-related IVs were extracted from GWAS for MR analysis. The summarized statistics of all correlated SNPs for *H. pylori* from the published GWASs are shown in Supplementary Table 2. The F-statistics for all SNPs were greater than 10, indicating a low risk of weak instrument bias.^27^

**Figure 2:**
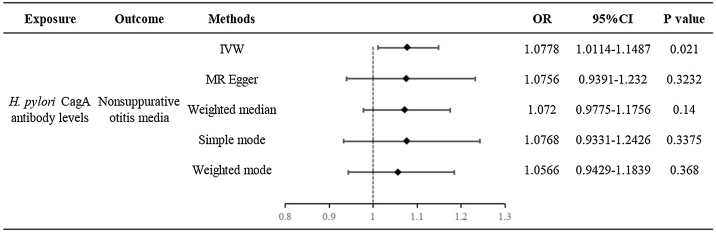
MR estimates the causal effect of *H. pylori* CagA antibodies on nonsuppurative otitis media based on each method.

According to the MR analysis results, we found that the levels of *H. pylori* CagA antibodies are associated with an increased risk of nonsuppurative otitis media (OR = 1.0778, 95% CI 1.0114–1.1487, *p*-value = 0.021) (Fig. 2). MR estimates of the causal effect of *H. pylori* infection on otolaryngological diseases based on each method are provided in Supplementary Table 3.

Sensitivity analyses showed that there was no evidence of potential heterogeneity or horizontal pleiotropy (Supplementary Table 4). In both IVW and MR-Egger regression tests for heterogeneity, the *p-*value of the Cochran Q statistic exceeded 0.05. Additionally, the IVs did not show any evidence of horizontal pleiotropy based on the MR-Egger and MR-PRESSO tests. Individual SNPs were utilized as IVs, and the funnel plot exhibits a symmetrical distribution of dots, suggesting that potential bias was unlikely to influence the causal relationship significantly. Leave-one-out analysis showed that the causal estimates of exposure and outcomes were not driven by any individual SNP. Leave-one-out analysis, funnel plots, scatter plots, and MR Effect sizes of *H. pylori* CagA antibody levels on nonsuppurative otitis media are shown in Supplementary material.

## Discussion

In this study based on GWASs data, a two-sample MR method was employed to investigate the association between *H. pylori* infection and 12 common otolaryngological diseases. The results indicate that *H. pylori* infection increases the risk of non-suppurative otitis media, while there was no causal relationship with other 11 common otolaryngological diseases. Multiple sensitivity analyses confirmed the robustness of the MR results.

While *H. pylori* infection has long been associated with gastrointestinal diseases, its impact on otolaryngological diseases remains unclear. Previous research indicates that *H. pylori* is not only present in the nasal sinus mucosa,^13^ tonsils,^29^ and larynx,^30^ but also has been detected in the middle ear.^31^

OME is commonly regarded as a type of non-suppurative otitis media characterized by the formation of middle ear effusion. The relationship between *H. pylori* and OME has been investigated in many previous studies. It has been reported that the detection rate of *H. pylori* in the middle ear, tonsils, and gastric fluid of children with OME is higher than that of children without OME.^32^ A case-control study indicated that the detection rate of *H. pylori* in middle ear effusion was 70%, suggesting that *H. pylori* may be one of the important bacteria influencing the pathogenesis of effusion otitis media.^10^ Additionally, another meta-analysis including 11 studies on the relationship between *H. pylori* infection and OME risk found a positive correlation between *H. pylori* infection and OME risk in children.^33^ Furthermore, a randomized controlled trial showed that the efficacy of anti-*H. pylori* therapy (clarithromycin, metronidazole, lansoprazole) for children with OME was 68.7%, higher than that of the traditional treatment group (amoxicillin, clavulanic acid) at 49.3%. After a 4-week follow-up, the patients' fecal *H. pylori* turned completely negative, and there was a significant improvement in tympanometry and pure-tone hearing threshold.^34^ On the other hand, another meta-analysis indicated that eradication of *H. pylori* can alleviate symptoms of resistant OME.^32^ These data suggest that eradicating *H. pylori* may improve the cure rate of OME in children and could potentially be part of future OME treatments.

*H. pylori* primarily colonizes the mucosal side of gastric mucus, with a pH close to 7.0,^35^ while the pH of middle ear effusion ranges from 7.0 to 9.0.^10^ At the same time, mucin MUC5AC is considered the main receptor source for *H. pylori* colonization in gastric tissues.^36^ In chronic otitis media, the expression of mucin MUC5AC is significantly increased.^37^ This may contribute to the colonization of *H. pylori* in the middle ear.

Meanwhile, we did not observe an association between *H. pylori* infection and other otolaryngological diseases, which is inconsistent with some previous observational study results but aligns with certain epidemiological studies.^38^, ^39^ The emergence of such discrepancies may stem from the limitations inherent in observational research. Firstly, many studies of this kind are based on case-control and cross-sectional designs, which introduce uncertainty regarding temporal sequence and make it challenging to establish definitive causal relationships. Secondly, observational studies are susceptible to various confounding factors, which may bias the results. Thirdly, reverse causality may also impact the outcomes of observational studies. MR approaches are less susceptible to confounding bias or reverse causality because exposure estimation is based on the germline level, and genetic predictions of *H. pylori* infection for individuals exist before outcomes, thus strengthening the evidence for causal inference. Furthermore, all estimated SNPs for exposure and outcomes were derived from individuals of European ancestry, thereby reducing the bias induced by population stratification. Given its advantages, the MR method has become an effective and reliable method to investigate the relationship between *H. pylori* infection and otolaryngological diseases risk.

However, we also acknowledge the limitations of this study. Firstly, since the stringent threshold (*p* < 5 × 10^−8^) yielded minimal IVs, we opted for a more relaxed criterion (*p* < 5 × 10^−6^) for IV selection. Secondly, this study only included participants of European descent, which reduces the bias caused by population stratification while also limiting the generalizability of our research results in other ethnic groups. Thirdly, the levels of antibodies to *H. pylori* and actual ongoing infection may differ, as false-negative or false-positive results cannot be entirely ruled out. Despite these limitations, investigating the underlying mechanism linking *H. pylori* infection and the development of otolaryngological diseases remains a valuable endeavor. Therefore, future research endeavors should focus on validating our findings to elucidate the precise relationship between *H. pylori* and otolaryngological diseases, thereby furnishing more robust evidence for clinical practice.

## Conclusion

At the genetic level, this study provides evidence supporting the causal relationship between anti-*H. pylori* CagA levels and non-suppurative otitis media. Future research can further explore the mechanisms at these molecular levels to deepen our understanding of the development of otolaryngological diseases and provide more effective treatment methods for related conditions.

## Funding

This work was supported by the Natural Science Foundation of Sichuan Province (Grant nº 2023NSFSC0621) and the Chengdu Medical Research Project (Grant nº 2022013).

## Ethics approval and consent

This study only used publicly available GWAS summary data, so ethical approval is not required.

## Conflicts of interest

The authors declare no conflicts of interest.

## Availability of data and materials

Publicly available datasets were analyzed in this study. The data of anti- *Helicobacter pylori* antibodies and otolaryngological diseases can be obtained from https://gwas.mrcieu.ac.uk/.
