# Supplementary material for: Association between Helicobacter pylori antibodies and otolaryngological diseases
Source: Braz J Otorhinolaryngol. 2024 Aug 8;90(6):101488. doi: 10.1016/j.bjorl.2024.101488 (PMC11393579; doi:10.1016/j.bjorl.2024.101488)

BJORL-D-24-00187_Supplementary Material

**Supplementary Table 1** Information sources for exposure and outcomes phenotypes.

|  |  | **ID** | **nCase** | **nControl** | **Data source** | **Population** |
| --- | --- | --- | --- | --- | --- | --- |
| **Exposure** | Anti-helicobacter pylori IgG seropositivity | ebi-a-GCST90006910 |  |  | Consortium (Butler-Laporte et al.) | European |
|  | Helicobacter pylori CagA antibody levels | ebi-a-GCST90006911 |  |  | Consortium (Butler-Laporte et al.) | European |
|  | Helicobacter pylori Catalase antibody levels | ebi-a-GCST90006912 |  |  | Consortium (Butler-Laporte et al.) | European |
|  | Helicobacter pylori GroEL antibody levels | ebi-a-GCST90006913 |  |  | Consortium (Butler-Laporte et al.) | European |
|  | Helicobacter pylori OMP antibody levels | ebi-a-GCST90006914 |  |  | Consortium (Butler-Laporte et al.) | European |
|  | Helicobacter pylori UREA antibody levels | ebi-a-GCST90006915 |  |  | Consortium (Butler-Laporte et al.) | European |
|  | Helicobacter pylori VacA antibody levels | ebi-a-GCST90006916 |  |  | Consortium (Butler-Laporte et al.) | European |
| **Outcomes** | Acute upper respiratory infection | finn-b-J10_UPPERINFEC | 35.847 | 182.945 | The FinnGen consortium | European |
|  | Chronic diseases of tonsils and adenoids | finn-b-J10_CHRONTONSADEN | 24.463 | 167.849 | The FinnGen consortium | European |
|  | Chronic laryngitis and laryngotracheitis | finn-b-J10_CHRONLARYNGITIS | 2.138 | 167.849 | The FinnGen consortium | European |
|  | Acute sinusitis | finn-b-J10_SINUSITIS | 10.916 | 182.945 | The FinnGen consortium | European |
|  | Chronic sinusitis | finn-b-J10_CHRONSINUSITIS | 8.524 | 167.849 | The FinnGen consortium | European |
|  | Allergic rhinitis | ukb-b-7178 | 25.486 | 87.097 | MRC-IEU | European |
|  | Nasal polyps | finn-b-J10_NASALPOLYP | 3.236 | 167.849 | The FinnGen consortium | European |
|  | Acute suppurative otitis media | finn-b-H8_SUP_ACUTE | 5.232 | 205.939 | The FinnGen consortium | European |
|  | Chronic suppurative otitis media | ebi-a-GCST90018809 | 1.108 | 483.037 | Consortium (Sakaue et al.) | European |
|  | Nonsuppurative otitis media | finn-b-H8_NONSUPPNAS | 4381 | 205939 | The FinnGen consortium | European |
|  | Sleep apnoea | ukb-b-16781 | 2.320 | 460.690 | MRC-IEU | European |
|  | Laryngeal cancer | ieu-b-4913 | 273 | 372.016 | UK Biobank | European |

**Supplementary Table 2** Genetic variation SNPs of helicobacter pylori antibodies from GWASs.

| **Exposure** | **SNPs** | **Chr** | **POS** | **Effect Allele** | **Other Allele** | **EAF** | **β** | **Se** | ***p-*value** | **F statistic** |
| --- | --- | --- | --- | --- | --- | --- | --- | --- | --- | --- |
| Anti-helicobacter pylori IgG seropositivity | rs60696037 | 1 | 23082564 | T | C | 0.091749 | -0.0561058 | 0.0118003 | 1,99E-06 | 22,6063 |
| Anti-helicobacter pylori IgG seropositivity | rs681900 | 2 | 75074967 | C | T | 0.240756 | -0.0375028 | 0.00790877 | 2,12E-06 | 22,4859 |
| Anti-helicobacter pylori IgG seropositivity | rs10044474 | 5 | 67425603 | G | T | 0.06739 | 0.0653924 | 0.0137102 | 1,85E-06 | 22,7492 |
| Anti-helicobacter pylori IgG seropositivity | rs117192929 | 7 | 89122359 | G | A | 0.044981 | 0.0767204 | 0.0166915 | 4,30E-06 | 21,1267 |
| Anti-helicobacter pylori IgG seropositivity | rs17232730 | 8 | 129537746 | C | G | 0.121486 | -0.0521098 | 0.0104981 | 6,91E-07 | 24,6387 |
| Anti-helicobacter pylori IgG seropositivity | rs11201988 | 10 | 88113475 | A | G | 0.117772 | 0.0505729 | 0.0109411 | 3,80E-06 | 21,3655 |
| Anti-helicobacter pylori IgG seropositivity | rs111575036 | 10 | 45194547 | T | C | 0.018243 | -0.116546 | 0.0252727 | 4,00E-06 | 21,2663 |
| Anti-helicobacter pylori IgG seropositivity | rs74347185 | 12 | 75543807 | A | G | 0.052948 | 0.0780404 | 0.0151369 | 2,53E-07 | 26,5806 |
| Anti-helicobacter pylori IgG seropositivity | rs76753623 | 15 | 61751677 | G | A | 0.039382 | 0.0842701 | 0.0172103 | 9,76E-07 | 23,9756 |
| Anti-helicobacter pylori IgG seropositivity | rs9747624 | 17 | 1022796 | C | G | 0.048352 | 0.0838839 | 0.0179288 | 2,89E-06 | 21,8905 |
| Anti-helicobacter pylori IgG seropositivity | rs62195865 | 20 | 8239626 | C | T | 0.019004 | 0.117182 | 0.0247662 | 2,23E-06 | 22,3874 |
| Helicobacter pylori CagA antibody levels | rs116421363 | 1 | 181820561 | C | A | 0.363202 | -0.223345 | 0.0475812 | 2,68E-06 | 22,0334 |
| Helicobacter pylori CagA antibody levels | rs75170215 | 3 | 25908239 | C | T | 0.02057 | 0.743256 | 0.156416 | 2,02E-06 | 22,5795 |
| Helicobacter pylori CagA antibody levels | rs75740599 | 4 | 159123611 | A | G | 0.252886 | 0.235842 | 0.0512963 | 4,27E-06 | 21,1383 |
| Helicobacter pylori CagA antibody levels | rs3998182 | 6 | 32561915 | C | T | 0.27555 | 0.284762 | 0.0548049 | 2,04E-07 | 26,9976 |
| Helicobacter pylori CagA antibody levels | rs571061419 | 6 | 162760485 | A | G | 0.074197 | -0.392137 | 0.0852358 | 4,21E-06 | 21,1656 |
| Helicobacter pylori CagA antibody levels | rs6530847 | 8 | 15111565 | A | T | 0.391211 | -0.210266 | 0.0452734 | 3,41E-06 | 21,5701 |
| Helicobacter pylori CagA antibody levels | rs4268452 | 10 | 81319354 | T | C | 0.071203 | -0.384976 | 0.0840329 | 4,62E-06 | 20,9879 |
| Helicobacter pylori CagA antibody levels | rs117827497 | 11 | 105212623 | G | A | 0.030462 | -0.583731 | 0.125147 | 3,10E-06 | 21,7563 |
| Helicobacter pylori CagA antibody levels | rs138363822 | 11 | 1606147 | G | A | 0.426282 | 0.218333 | 0.0475038 | 4,30E-06 | 21,1243 |
| Helicobacter pylori CagA antibody levels | rs117537486 | 11 | 94351805 | C | G | 0.03072 | 0.596789 | 0.129736 | 4,22E-06 | 21,1602 |
| Helicobacter pylori CagA antibody levels | rs149747348 | 12 | 98890443 | C | G | 0.013186 | -0.895407 | 0.194003 | 3,92E-06 | 21,3022 |
| Helicobacter pylori CagA antibody levels | rs56264437 | 13 | 102123887 | A | G | 0.603666 | 0.233903 | 0.0491972 | 1,99E-06 | 22,6043 |
| Helicobacter pylori CagA antibody levels | rs11858369 | 15 | 90495509 | G | A | 0.068381 | 0.437721 | 0.0864978 | 4,18E-07 | 25,6085 |
| Helicobacter pylori CagA antibody levels | rs553266653 | 16 | 46420007 | C | A | 0.033679 | -0.731511 | 0.158708 | 4,04E-06 | 21,2444 |
| Helicobacter pylori CagA antibody levels | rs118006294 | 16 | 10974221 | C | T | 0.050367 | -0.475295 | 0.101815 | 3,04E-06 | 21,7923 |
| Helicobacter pylori Catalase antibody levels | rs190890483 | 2 | 157083346 | A | G | 0.012211 | -0.771796 | 0.156854 | 8,63E-07 | 24,2111 |
| Helicobacter pylori Catalase antibody levels | rs72808426 | 2 | 58133048 | T | C | 0.035007 | -0.433914 | 0.0938646 | 3,79E-06 | 21,37 |
| Helicobacter pylori Catalase antibody levels | rs58679787 | 2 | 16452055 | T | C | 0.01117 | 0.776521 | 0.168076 | 3,84E-06 | 21,3449 |
| Helicobacter pylori Catalase antibody levels | rs343686 | 6 | 80607062 | C | A | 0.620724 | -0.174703 | 0.036546 | 1,75E-06 | 22,8518 |
| Helicobacter pylori Catalase antibody levels | rs6456714 | 6 | 26335346 | G | C | 0.631631 | 0.170902 | 0.0360891 | 2,18E-06 | 22,4255 |
| Helicobacter pylori Catalase antibody levels | rs77162569 | 7 | 148264987 | T | C | 0.025229 | -0.555536 | 0.10878 | 3,27E-07 | 26,0811 |
| Helicobacter pylori Catalase antibody levels | rs17647677 | 13 | 46749781 | C | T | 0.041284 | -0.394124 | 0.0851199 | 3,65E-06 | 21,439 |
| Helicobacter pylori Catalase antibody levels | rs927062 | 14 | 33095049 | G | A | 0.213718 | -0.197935 | 0.0421097 | 2,60E-06 | 22,0943 |
| Helicobacter pylori Catalase antibody levels | rs147016571 | 16 | 25837192 | G | A | 0.010184 | -0.839947 | 0.175759 | 1,76E-06 | 22,8386 |
| Helicobacter pylori GroEL antibody levels | rs3104037 | 6 | 107139657 | T | C | 0.613619 | 0.134138 | 0.0281607 | 1,90E-06 | 22,6891 |
| Helicobacter pylori GroEL antibody levels | rs61680606 | 10 | 128939945 | C | A | 0.225838 | 0.152271 | 0.0322746 | 2,38E-06 | 22,2594 |
| Helicobacter pylori GroEL antibody levels | rs7099832 | 10 | 130790371 | C | G | 0.4356 | -0.133798 | 0.0276583 | 1,31E-06 | 23,4017 |
| Helicobacter pylori GroEL antibody levels | rs117341694 | 13 | 66889675 | A | G | 0.013029 | 0.54338 | 0.115856 | 2,73E-06 | 21,9973 |
| Helicobacter pylori GroEL antibody levels | rs1367344 | 18 | 71479072 | C | T | 0.223984 | -0.155199 | 0.0330617 | 2,68E-06 | 22,0357 |
| Helicobacter pylori OMP antibody levels | rs703135 | 1 | 158678095 | A | G | 0.417825 | -0.133184 | 0.0280688 | 2,09E-06 | 22,5142 |
| Helicobacter pylori OMP antibody levels | rs1156822 | 2 | 41824828 | T | A | 0.543158 | -0.127607 | 0.0277813 | 4,36E-06 | 21,0981 |
| Helicobacter pylori OMP antibody levels | rs55862931 | 2 | 2672878 | C | G | 0.258412 | -0.146339 | 0.0316679 | 3,82E-06 | 21,3541 |
| Helicobacter pylori OMP antibody levels | rs143477841 | 2 | 24042805 | A | G | 0.012119 | -0.572525 | 0.124655 | 4,37E-06 | 21,0945 |
| Helicobacter pylori OMP antibody levels | rs9276733 | 6 | 32766026 | G | A | 0.229896 | 0.195698 | 0.0364829 | 8,14E-08 | 28,7736 |
| Helicobacter pylori OMP antibody levels | rs3104361 | 6 | 32652278 | C | T | 0.581879 | 0.181489 | 0.0293752 | 6,48E-10 | 38,1715 |
| Helicobacter pylori OMP antibody levels | rs60949128 | 8 | 41936404 | A | C | 0.064465 | -0.277296 | 0.0563277 | 8,53E-07 | 24,235 |
| Helicobacter pylori OMP antibody levels | rs116944686 | 8 | 101374808 | A | G | 0.023319 | 0.417806 | 0.0913422 | 4,78E-06 | 20,9222 |
| Helicobacter pylori OMP antibody levels | rs8019346 | 14 | 65353011 | A | G | 0.08492 | 0.229272 | 0.0498505 | 4,24E-06 | 21,1526 |
| Helicobacter pylori OMP antibody levels | rs1892331 | 20 | 59326926 | G | A | 0.032321 | 0.376964 | 0.0761458 | 7,40E-07 | 24,508 |
| Helicobacter pylori UREA antibody levels | rs9289888 | 3 | 152849257 | T | C | 0.066303 | 0.272084 | 0.0573847 | 2,12E-06 | 22,4809 |
| Helicobacter pylori UREA antibody levels | rs4689912 | 4 | 4659633 | C | T | 0.267281 | -0.158009 | 0.0331663 | 1,90E-06 | 22,6971 |
| Helicobacter pylori UREA antibody levels | rs71569678 | 6 | 23380648 | C | A | 0.060345 | 0.340169 | 0.0613821 | 2,99E-08 | 30,7119 |
| Helicobacter pylori UREA antibody levels | rs75477465 | 8 | 68794331 | A | G | 0.034029 | 0.374873 | 0.0802966 | 3,03E-06 | 21,7959 |
| Helicobacter pylori UREA antibody levels | rs61907106 | 12 | 4003258 | A | G | 0.048196 | -0.358847 | 0.0702596 | 3,27E-07 | 26,086 |
| Helicobacter pylori UREA antibody levels | rs12820262 | 12 | 129244638 | A | G | 0.177117 | 0.206073 | 0.0393175 | 1,59E-07 | 27,4707 |
| Helicobacter pylori UREA antibody levels | rs60386004 | 14 | 80136052 | T | A | 0.158161 | 0.191768 | 0.0407276 | 2,49E-06 | 22,1705 |
| Helicobacter pylori UREA antibody levels | rs143570118 | 17 | 76370080 | T | C | 0.029208 | 0.434126 | 0.0926047 | 2,76E-06 | 21,9769 |
| Helicobacter pylori UREA antibody levels | rs117994655 | 19 | 8188056 | A | G | 0.032509 | 0.402018 | 0.0828335 | 1,21E-06 | 23,5548 |
| Helicobacter pylori UREA antibody levels | rs134748 | 22 | 26543287 | T | G | 0.653005 | -0.142202 | 0.0306515 | 3,50E-06 | 21,5233 |
| Helicobacter pylori VacA antibody levels | rs113063793 | 1 | 217309978 | T | A | 0.056242 | 0.373402 | 0.075648 | 7,97E-07 | 24,3645 |
| Helicobacter pylori VacA antibody levels | rs72645538 | 1 | 9453174 | G | A | 0.011992 | 0.752792 | 0.164377 | 4,66E-06 | 20,9734 |
| Helicobacter pylori VacA antibody levels | rs113845906 | 3 | 94372336 | A | G | 0.045066 | 0.413478 | 0.0845187 | 9,97E-07 | 23,9331 |
| Helicobacter pylori VacA antibody levels | rs1530121 | 5 | 124624902 | C | T | 0.86313 | -0.266111 | 0.0511414 | 1,96E-07 | 27,0757 |
| Helicobacter pylori VacA antibody levels | rs372744619 | 6 | 32434141 | G | A | 0.010519 | 0.887411 | 0.173802 | 3,29E-07 | 26,0699 |
| Helicobacter pylori VacA antibody levels | rs77497849 | 7 | 32049959 | G | A | 0.119223 | 0.264227 | 0.0538191 | 9,13E-07 | 24,1036 |
| Helicobacter pylori VacA antibody levels | rs10246445 | 7 | 2981123 | C | A | 0.028197 | -0.488144 | 0.106857 | 4,92E-06 | 20,8684 |
| Helicobacter pylori VacA antibody levels | rs7019543 | 9 | 110365310 | T | G | 0.320078 | 0.18107 | 0.0380994 | 2,01E-06 | 22,5869 |
| Helicobacter pylori VacA antibody levels | rs59104649 | 10 | 129150690 | T | A | 0.014016 | 0.68347 | 0.147207 | 3,44E-06 | 21,5567 |
| Helicobacter pylori VacA antibody levels | rs117077218 | 10 | 90083333 | T | C | 0.016656 | 0.640222 | 0.138755 | 3,95E-06 | 21,2894 |
| Helicobacter pylori VacA antibody levels | rs148556020 | 12 | 28904914 | A | T | 0.016883 | 0.63085 | 0.137457 | 4,44E-06 | 21,0629 |
| Helicobacter pylori VacA antibody levels | rs11044935 | 12 | 20062085 | T | A | 0.039367 | 0.428278 | 0.0905091 | 2,22E-06 | 22,3907 |
| Helicobacter pylori VacA antibody levels | rs73500239 | 16 | 1332341 | C | G | 0.144805 | 0.236088 | 0.0500401 | 2,38E-06 | 22,2593 |
| Helicobacter pylori VacA antibody levels | rs9606224 | 22 | 20034187 | T | C | 0.029841 | 0.507461 | 0.10223 | 6,91E-07 | 24,6404 |
| Helicobacter pylori VacA antibody levels | rs133537 | 22 | 48595971 | C | T | 0.615079 | -0.173609 | 0.0367088 | 2,25E-06 | 22,3668 |

SNP, Single Nucleus Polymorphism; GWASs, Genome-Wide Association Studies.

**Supplementary Table 3** MR estimates of helicobacter pylori antibody's causal effect on outcomes based on each method.

| **Exposure** | **Outcomes** | **Method** | **nSNP** | **OR** | **p** | **95% CI** |
| --- | --- | --- | --- | --- | --- | --- |
| Anti-helicobacter pylori IgG seropositivity | Acute upper respiratory infections | Inverse variance weighted | 10 | 1.0604 | 0.4672 | 0.9053‒1.2421 |
| Anti-helicobacter pylori IgG seropositivity | Acute upper respiratory infections | MR Egger | 10 | 1.152 | 0.6526 | 0.6366‒2.0849 |
| Anti-helicobacter pylori IgG seropositivity | Acute upper respiratory infections | Weighted median | 10 | 1.0283 | 0.8007 | 0.828‒1.277 |
| Anti-helicobacter pylori IgG seropositivity | Acute upper respiratory infections | Simple mode | 10 | 1.0419 | 0.8142 | 0.7475‒1.4522 |
| Anti-helicobacter pylori IgG seropositivity | Acute upper respiratory infections | Weighted mode | 10 | 1.049 | 0.7664 | 0.7723‒1.4248 |
| Helicobacter pylori CagA antibody levels | Acute upper respiratory infections | Inverse variance weighted | 10 | 1.0092 | 0.6042 | 0.9749‒1.0446 |
| Helicobacter pylori CagA antibody levels | Acute upper respiratory infections | MR Egger | 10 | 1.0352 | 0.3951 | 0.9599‒1.1164 |
| Helicobacter pylori CagA antibody levels | Acute upper respiratory infections | Weighted median | 10 | 1.0272 | 0.1555 | 0.9899‒1.0659 |
| Helicobacter pylori CagA antibody levels | Acute upper respiratory infections | Simple mode | 10 | 1.0419 | 0.205 | 0.9823‒1.105 |
| Helicobacter pylori CagA antibody levels | Acute upper respiratory infections | Weighted mode | 10 | 1.0338 | 0.1731 | 0.9893‒1.0803 |
| Helicobacter pylori Catalase antibody levels | Acute upper respiratory infections | Inverse variance weighted | 8 | 0.985 | 0.3664 | 0.9532‒1.0179 |
| Helicobacter pylori Catalase antibody levels | Acute upper respiratory infections | MR Egger | 8 | 0.9773 | 0.4862 | 0.9198‒1.0384 |
| Helicobacter pylori Catalase antibody levels | Acute upper respiratory infections | Weighted median | 8 | 0.9736 | 0.2257 | 0.9323‒1.0167 |
| Helicobacter pylori Catalase antibody levels | Acute upper respiratory infections | Simple mode | 8 | 0.9602 | 0.2536 | 0.9007‒1.0237 |
| Helicobacter pylori Catalase antibody levels | Acute upper respiratory infections | Weighted mode | 8 | 0.9673 | 0.272 | 0.9158‒1.0217 |
| Helicobacter pylori GroEL antibody levels | Acute upper respiratory infections | Inverse variance weighted | 3 | 0.9999 | 0.9981 | 0.8981‒1.1131 |
| Helicobacter pylori GroEL antibody levels | Acute upper respiratory infections | MR Egger | 3 | 0.8589 | 0.316 | 0.7309‒1.0094 |
| Helicobacter pylori GroEL antibody levels | Acute upper respiratory infections | Weighted median | 3 | 1.0333 | 0.5359 | 0.9315‒1.1463 |
| Helicobacter pylori GroEL antibody levels | Acute upper respiratory infections | Simple mode | 3 | 1.0626 | 0.483 | 0.9244‒1.2214 |
| Helicobacter pylori GroEL antibody levels | Acute upper respiratory infections | Weighted mode | 3 | 1.0626 | 0.4741 | 0.9274‒1.2175 |
| Helicobacter pylori OMP antibody levels | Acute upper respiratory infections | Inverse variance weighted | 8 | 1.0029 | 0.9219 | 0.9472‒1.0617 |
| Helicobacter pylori OMP antibody levels | Acute upper respiratory infections | MR Egger | 8 | 1.0823 | 0.4507 | 0.8931‒1.3116 |
| Helicobacter pylori OMP antibody levels | Acute upper respiratory infections | Weighted median | 8 | 1.0113 | 0.7533 | 0.9428‒1.0849 |
| Helicobacter pylori OMP antibody levels | Acute upper respiratory infections | Simple mode | 8 | 1.0003 | 0.9965 | 0.8907‒1.1234 |
| Helicobacter pylori OMP antibody levels | Acute upper respiratory infections | Weighted mode | 8 | 1.0197 | 0.6942 | 0.9289‒1.1194 |
| Helicobacter pylori UREA antibody levels | Acute upper respiratory infections | Inverse variance weighted | 10 | 1.0266 | 0.1719 | 0.9887‒1.066 |
| Helicobacter pylori UREA antibody levels | Acute upper respiratory infections | MR Egger | 10 | 1.0701 | 0.2402 | 0.9637‒1.1883 |
| Helicobacter pylori UREA antibody levels | Acute upper respiratory infections | Weighted median | 10 | 1.0256 | 0.3124 | 0.9765‒1.0771 |
| Helicobacter pylori UREA antibody levels | Acute upper respiratory infections | Simple mode | 10 | 1.019 | 0.6357 | 0.945‒1.0989 |
| Helicobacter pylori UREA antibody levels | Acute upper respiratory infections | Weighted mode | 10 | 1.0202 | 0.5882 | 0.9513‒1.0941 |
| Helicobacter pylori VacA antibody levels | Acute upper respiratory infections | Inverse variance weighted | 13 | 0.9786 | 0.112 | 0.9529‒1.0051 |
| Helicobacter pylori VacA antibody levels | Acute upper respiratory infections | MR Egger | 13 | 0.9831 | 0.5344 | 0.9331‒1.0357 |
| Helicobacter pylori VacA antibody levels | Acute upper respiratory infections | Weighted median | 13 | 0.9836 | 0.3709 | 0.9486‒1.0199 |
| Helicobacter pylori VacA antibody levels | Acute upper respiratory infections | Simple mode | 13 | 0.9818 | 0.5897 | 0.9199‒1.0478 |
| Helicobacter pylori VacA antibody levels | Acute upper respiratory infections | Weighted mode | 13 | 0.9847 | 0.6207 | 0.9279‒1.045 |
| Anti-helicobacter pylori IgG seropositivity | Chronic diseases of tonsils and adenoids | Inverse variance weighted | 10 | 1.0361 | 0.7317 | 0.776‒1.3834 |
| Anti-helicobacter pylori IgG seropositivity | Chronic diseases of tonsils and adenoids | MR Egger | 10 | 1.2804 | 0.6259 | 0.4362‒3.7583 |
| Anti-helicobacter pylori IgG seropositivity | Chronic diseases of tonsils and adenoids | Weighted median | 10 | 1.1502 | 0.4477 | 0.8686‒1.523 |
| Anti-helicobacter pylori IgG seropositivity | Chronic diseases of tonsils and adenoids | Simple mode | 10 | 1.1996 | 0.5694 | 0.7683‒1.8729 |
| Anti-helicobacter pylori IgG seropositivity | Chronic diseases of tonsils and adenoids | Weighted mode | 10 | 1.1909 | 0.5102 | 0.8296‒1.7096 |
| Helicobacter pylori CagA antibody levels | Chronic diseases of tonsils and adenoids | Inverse variance weighted | 10 | 0.9751 | 0.7495 | 0.9453‒1.0058 |
| Helicobacter pylori CagA antibody levels | Chronic diseases of tonsils and adenoids | MR Egger | 10 | 0.9879 | 0.2111 | 0.9248‒1.0554 |
| Helicobacter pylori CagA antibody levels | Chronic diseases of tonsils and adenoids | Weighted median | 10 | 0.9874 | 0.497 | 0.9463‒1.0303 |
| Helicobacter pylori CagA antibody levels | Chronic diseases of tonsils and adenoids | Simple mode | 10 | 0.9989 | 0.6654 | 0.9355‒1.0665 |
| Helicobacter pylori CagA antibody levels | Chronic diseases of tonsils and adenoids | Weighted mode | 10 | 0.9958 | 0.6828 | 0.942‒1.0528 |
| Helicobacter pylori Catalase antibody levels | Chronic diseases of tonsils and adenoids | Inverse variance weighted | 8 | 0.9832 | 0.1999 | 0.942‒1.0262 |
| Helicobacter pylori Catalase antibody levels | Chronic diseases of tonsils and adenoids | MR Egger | 8 | 1.0344 | 0.0455 | 0.9594‒1.1153 |
| Helicobacter pylori Catalase antibody levels | Chronic diseases of tonsils and adenoids | Weighted median | 8 | 0.9907 | 0.8146 | 0.9364‒1.0482 |
| Helicobacter pylori Catalase antibody levels | Chronic diseases of tonsils and adenoids | Simple mode | 8 | 0.9841 | 0.638 | 0.9065‒1.0685 |
| Helicobacter pylori Catalase antibody levels | Chronic diseases of tonsils and adenoids | Weighted mode | 8 | 0.9859 | 0.6208 | 0.927‒1.0485 |
| Helicobacter pylori GroEL antibody levels | Chronic diseases of tonsils and adenoids | Inverse variance weighted | 3 | 0.9938 | 0.6022 | 0.9073‒1.0886 |
| Helicobacter pylori GroEL antibody levels | Chronic diseases of tonsils and adenoids | MR Egger | 3 | 1.0378 | 0.9377 | 0.8495‒1.2678 |
| Helicobacter pylori GroEL antibody levels | Chronic diseases of tonsils and adenoids | Weighted median | 3 | 0.9928 | 0.8837 | 0.893‒1.1036 |
| Helicobacter pylori GroEL antibody levels | Chronic diseases of tonsils and adenoids | Simple mode | 3 | 0.982 | 0.8189 | 0.8694‒1.1093 |
| Helicobacter pylori GroEL antibody levels | Chronic diseases of tonsils and adenoids | Weighted mode | 3 | 0.9897 | 0.8415 | 0.8714‒1.1241 |
| Helicobacter pylori OMP antibody levels | Chronic diseases of tonsils and adenoids | Inverse variance weighted | 8 | 0.9471 | 0.2009 | 0.8839‒1.0149 |
| Helicobacter pylori OMP antibody levels | Chronic diseases of tonsils and adenoids | MR Egger | 8 | 0.8572 | 0.4779 | 0.6809‒1.0792 |
| Helicobacter pylori OMP antibody levels | Chronic diseases of tonsils and adenoids | Weighted median | 8 | 0.9618 | 0.0692 | 0.8879‒1.0419 |
| Helicobacter pylori OMP antibody levels | Chronic diseases of tonsils and adenoids | Simple mode | 8 | 0.9543 | 0.1905 | 0.8596‒1.0593 |
| Helicobacter pylori OMP antibody levels | Chronic diseases of tonsils and adenoids | Weighted mode | 8 | 0.9576 | 0.2012 | 0.8713‒1.0524 |
| Helicobacter pylori UREA antibody levels | Chronic diseases of tonsils and adenoids | Inverse variance weighted | 10 | 0.9768 | 0.5627 | 0.9278‒1.0283 |
| Helicobacter pylori UREA antibody levels | Chronic diseases of tonsils and adenoids | MR Egger | 10 | 1.0568 | 0.6792 | 0.9189‒1.2153 |
| Helicobacter pylori UREA antibody levels | Chronic diseases of tonsils and adenoids | Weighted median | 10 | 0.9521 | 0.1397 | 0.8909‒1.0175 |
| Helicobacter pylori UREA antibody levels | Chronic diseases of tonsils and adenoids | Simple mode | 10 | 0.9791 | 0.2773 | 0.8833‒1.0852 |
| Helicobacter pylori UREA antibody levels | Chronic diseases of tonsils and adenoids | Weighted mode | 10 | 0.9597 | 0.2882 | 0.8721‒1.056 |
| Helicobacter pylori VacA antibody levels | Chronic diseases of tonsils and adenoids | Inverse variance weighted | 13 | 0.9777 | 0.1734 | 0.944‒1.0126 |
| Helicobacter pylori VacA antibody levels | Chronic diseases of tonsils and adenoids | MR Egger | 13 | 0.96 | 0.9135 | 0.8945‒1.0302 |
| Helicobacter pylori VacA antibody levels | Chronic diseases of tonsils and adenoids | Weighted median | 13 | 0.9656 | 0.6002 | 0.9227‒1.0105 |
| Helicobacter pylori VacA antibody levels | Chronic diseases of tonsils and adenoids | Simple mode | 13 | 0.9336 | 0.7544 | 0.8601‒1.0132 |
| Helicobacter pylori VacA antibody levels | Chronic diseases of tonsils and adenoids | Weighted mode | 13 | 0.9622 | 0.7478 | 0.9027‒1.0255 |
| Anti-helicobacter pylori IgG seropositivity | Chronic laryngitis and laryngotracheitis | Inverse variance weighted | 10 | 1.0841 | 0.8173 | 0.5466‒2.1499 |
| Anti-helicobacter pylori IgG seropositivity | Chronic laryngitis and laryngotracheitis | MR Egger | 10 | 0.9335 | 0.9592 | 0.0727‒11.9841 |
| Anti-helicobacter pylori IgG seropositivity | Chronic laryngitis and laryngotracheitis | Weighted median | 10 | 1.7598 | 0.1321 | 0.8433‒3.6728 |
| Anti-helicobacter pylori IgG seropositivity | Chronic laryngitis and laryngotracheitis | Simple mode | 10 | 2.3615 | 0.1878 | 0.7244‒7.6987 |
| Anti-helicobacter pylori IgG seropositivity | Chronic laryngitis and laryngotracheitis | Weighted mode | 10 | 2.2166 | 0.1977 | 0.722‒6.8055 |
| Helicobacter pylori CagA antibody levels | Chronic laryngitis and laryngotracheitis | Inverse variance weighted | 10 | 0.9594 | 0.3622 | 0.8776‒1.0488 |
| Helicobacter pylori CagA antibody levels | Chronic laryngitis and laryngotracheitis | MR Egger | 10 | 0.8758 | 0.2089 | 0.7241‒1.0593 |
| Helicobacter pylori CagA antibody levels | Chronic laryngitis and laryngotracheitis | Weighted median | 10 | 0.9182 | 0.1702 | 0.8127‒1.0373 |
| Helicobacter pylori CagA antibody levels | Chronic laryngitis and laryngotracheitis | Simple mode | 10 | 0.9297 | 0.4852 | 0.764‒1.1314 |
| Helicobacter pylori CagA antibody levels | Chronic laryngitis and laryngotracheitis | Weighted mode | 10 | 0.9261 | 0.3406 | 0.7975‒1.0755 |
| Helicobacter pylori Catalase antibody levels | Chronic laryngitis and laryngotracheitis | Inverse variance weighted | 8 | 0.8928 | 0.059 | 0.7936‒1.0043 |
| Helicobacter pylori Catalase antibody levels | Chronic laryngitis and laryngotracheitis | MR Egger | 8 | 0.9399 | 0.596 | 0.7564‒1.1679 |
| Helicobacter pylori Catalase antibody levels | Chronic laryngitis and laryngotracheitis | Weighted median | 8 | 0.8553 | 0.0422 | 0.7356‒0.9945 |
| Helicobacter pylori Catalase antibody levels | Chronic laryngitis and laryngotracheitis | Simple mode | 8 | 0.8612 | 0.2213 | 0.6925‒1.0711 |
| Helicobacter pylori Catalase antibody levels | Chronic laryngitis and laryngotracheitis | Weighted mode | 8 | 0.868 | 0.1645 | 0.7259‒1.0379 |
| Helicobacter pylori GroEL antibody levels | Chronic laryngitis and laryngotracheitis | Inverse variance weighted | 3 | 0.9358 | 0.6263 | 0.7164‒1.2223 |
| Helicobacter pylori GroEL antibody levels | Chronic laryngitis and laryngotracheitis | MR Egger | 3 | 1.1435 | 0.7386 | 0.6252‒2.0916 |
| Helicobacter pylori GroEL antibody levels | Chronic laryngitis and laryngotracheitis | Weighted median | 3 | 0.9801 | 0.9041 | 0.7072‒1.3585 |
| Helicobacter pylori GroEL antibody levels | Chronic laryngitis and laryngotracheitis | Simple mode | 3 | 1.0119 | 0.9613 | 0.6626‒1.5454 |
| Helicobacter pylori GroEL antibody levels | Chronic laryngitis and laryngotracheitis | Weighted mode | 3 | 1.0208 | 0.9331 | 0.6669‒1.5627 |
| Helicobacter pylori OMP antibody levels | Chronic laryngitis and laryngotracheitis | Inverse variance weighted | 8 | 0.9818 | 0.8438 | 0.818‒1.1784 |
| Helicobacter pylori OMP antibody levels | Chronic laryngitis and laryngotracheitis | MR Egger | 8 | 0.6728 | 0.2167 | 0.3833‒1.181 |
| Helicobacter pylori OMP antibody levels | Chronic laryngitis and laryngotracheitis | Weighted median | 8 | 0.9348 | 0.5844 | 0.7343‒1.1901 |
| Helicobacter pylori OMP antibody levels | Chronic laryngitis and laryngotracheitis | Simple mode | 8 | 0.9774 | 0.9077 | 0.6738‒1.4179 |
| Helicobacter pylori OMP antibody levels | Chronic laryngitis and laryngotracheitis | Weighted mode | 8 | 1.0985 | 0.5944 | 0.7896‒1.5283 |
| Helicobacter pylori UREA antibody levels | Chronic laryngitis and laryngotracheitis | Inverse variance weighted | 10 | 0.9742 | 0.7233 | 0.8431‒1.1257 |
| Helicobacter pylori UREA antibody levels | Chronic laryngitis and laryngotracheitis | MR Egger | 10 | 1.5186 | 0.0584 | 1.0477‒2.2011 |
| Helicobacter pylori UREA antibody levels | Chronic laryngitis and laryngotracheitis | Weighted median | 10 | 0.9723 | 0.7725 | 0.8035‒1.1765 |
| Helicobacter pylori UREA antibody levels | Chronic laryngitis and laryngotracheitis | Simple mode | 10 | 0.9744 | 0.8634 | 0.7313‒1.2984 |
| Helicobacter pylori UREA antibody levels | Chronic laryngitis and laryngotracheitis | Weighted mode | 10 | 0.9744 | 0.8491 | 0.7518‒1.2629 |
| Helicobacter pylori VacA antibody levels | Chronic laryngitis and laryngotracheitis | Inverse variance weighted | 13 | 0.9617 | 0.5617 | 0.843‒1.0972 |
| Helicobacter pylori VacA antibody levels | Chronic laryngitis and laryngotracheitis | MR Egger | 13 | 0.906 | 0.4823 | 0.6945‒1.1821 |
| Helicobacter pylori VacA antibody levels | Chronic laryngitis and laryngotracheitis | Weighted median | 13 | 0.902 | 0.1533 | 0.783‒1.0392 |
| Helicobacter pylori VacA antibody levels | Chronic laryngitis and laryngotracheitis | Simple mode | 13 | 0.8473 | 0.1365 | 0.6912‒1.0385 |
| Helicobacter pylori VacA antibody levels | Chronic laryngitis and laryngotracheitis | Weighted mode | 13 | 0.865 | 0.1453 | 0.7207‒1.0382 |
| Anti-helicobacter pylori IgG seropositivity | Acute sinusitis | Inverse variance weighted | 10 | 0.9229 | 0.633 | 0.6639‒1.2829 |
| Anti-helicobacter pylori IgG seropositivity | Acute sinusitis | MR Egger | 10 | 0.9015 | 0.874 | 0.2608‒3.1166 |
| Anti-helicobacter pylori IgG seropositivity | Acute sinusitis | Weighted median | 10 | 1.0978 | 0.6208 | 0.7585‒1.5888 |
| Anti-helicobacter pylori IgG seropositivity | Acute sinusitis | Simple mode | 10 | 1.3066 | 0.4737 | 0.6482‒2.6336 |
| Anti-helicobacter pylori IgG seropositivity | Acute sinusitis | Weighted mode | 10 | 1.2784 | 0.5143 | 0.6291‒2.5978 |
| Helicobacter pylori CagA antibody levels | Acute sinusitis | Inverse variance weighted | 10 | 0.9957 | 0.8375 | 0.9556‒1.0375 |
| Helicobacter pylori CagA antibody levels | Acute sinusitis | MR Egger | 10 | 0.9786 | 0.641 | 0.8966‒1.0681 |
| Helicobacter pylori CagA antibody levels | Acute sinusitis | Weighted median | 10 | 0.9724 | 0.3172 | 0.9204‒1.0273 |
| Helicobacter pylori CagA antibody levels | Acute sinusitis | Simple mode | 10 | 0.9551 | 0.2871 | 0.882‒1.0342 |
| Helicobacter pylori CagA antibody levels | Acute sinusitis | Weighted mode | 10 | 0.9617 | 0.3117 | 0.8955‒1.0329 |
| Helicobacter pylori Catalase antibody levels | Acute sinusitis | Inverse variance weighted | 8 | 0.9763 | 0.5617 | 0.9002‒1.0587 |
| Helicobacter pylori Catalase antibody levels | Acute sinusitis | MR Egger | 8 | 0.9079 | 0.2425 | 0.7845‒1.0507 |
| Helicobacter pylori Catalase antibody levels | Acute sinusitis | Weighted median | 8 | 0.9422 | 0.1541 | 0.8682‒1.0226 |
| Helicobacter pylori Catalase antibody levels | Acute sinusitis | Simple mode | 8 | 0.9485 | 0.4854 | 0.824‒1.0918 |
| Helicobacter pylori Catalase antibody levels | Acute sinusitis | Weighted mode | 8 | 0.9323 | 0.1609 | 0.8542‒1.0177 |
| Helicobacter pylori GroEL antibody levels | Acute sinusitis | Inverse variance weighted | 3 | 1.0327 | 0.6532 | 0.8974‒1.1886 |
| Helicobacter pylori GroEL antibody levels | Acute sinusitis | MR Egger | 3 | 0.8698 | 0.4899 | 0.6675‒1.1336 |
| Helicobacter pylori GroEL antibody levels | Acute sinusitis | Weighted median | 3 | 1.001 | 0.9899 | 0.8556‒1.1712 |
| Helicobacter pylori GroEL antibody levels | Acute sinusitis | Simple mode | 3 | 0.9857 | 0.904 | 0.8008‒1.2132 |
| Helicobacter pylori GroEL antibody levels | Acute sinusitis | Weighted mode | 3 | 0.9755 | 0.8238 | 0.805‒1.1821 |
| Helicobacter pylori OMP antibody levels | Acute sinusitis | Inverse variance weighted | 8 | 1.1444 | 0.107 | 0.9713‒1.3485 |
| Helicobacter pylori OMP antibody levels | Acute sinusitis | MR Egger | 8 | 1.0797 | 0.8043 | 0.6042‒1.9294 |
| Helicobacter pylori OMP antibody levels | Acute sinusitis | Weighted median | 8 | 1.0669 | 0.3554 | 0.93‒1.2238 |
| Helicobacter pylori OMP antibody levels | Acute sinusitis | Simple mode | 8 | 0.9391 | 0.575 | 0.7617‒1.1579 |
| Helicobacter pylori OMP antibody levels | Acute sinusitis | Weighted mode | 8 | 0.9709 | 0.8282 | 0.751‒1.2553 |
| Helicobacter pylori UREA antibody levels | Acute sinusitis | Inverse variance weighted | 10 | 1.0113 | 0.7651 | 0.9394‒1.0888 |
| Helicobacter pylori UREA antibody levels | Acute sinusitis | MR Egger | 10 | 1.1551 | 0.1832 | 0.9514‒1.4025 |
| Helicobacter pylori UREA antibody levels | Acute sinusitis | Weighted median | 10 | 1.0007 | 0.9882 | 0.9159‒1.0933 |
| Helicobacter pylori UREA antibody levels | Acute sinusitis | Simple mode | 10 | 1.0117 | 0.8874 | 0.8649‒1.1834 |
| Helicobacter pylori UREA antibody levels | Acute sinusitis | Weighted mode | 10 | 1.0249 | 0.7701 | 0.8735‒1.2025 |
| Helicobacter pylori VacA antibody levels | Acute sinusitis | Inverse variance weighted | 13 | 0.9688 | 0.1569 | 0.9272‒1.0123 |
| Helicobacter pylori VacA antibody levels | Acute sinusitis | MR Egger | 13 | 0.9748 | 0.5728 | 0.8946‒1.0623 |
| Helicobacter pylori VacA antibody levels | Acute sinusitis | Weighted median | 13 | 0.9669 | 0.2607 | 0.9118‒1.0253 |
| Helicobacter pylori VacA antibody levels | Acute sinusitis | Simple mode | 13 | 1.0049 | 0.9154 | 0.9198‒1.0979 |
| Helicobacter pylori VacA antibody levels | Acute sinusitis | Weighted mode | 13 | 0.9792 | 0.5728 | 0.9122‒1.0512 |
| Anti-helicobacter pylori IgG seropositivity | Chronic sinusitis | Inverse variance weighted | 10 | 1.0418 | 0.813 | 0.7418‒1.4633 |
| Anti-helicobacter pylori IgG seropositivity | Chronic sinusitis | MR Egger | 10 | 1.8811 | 0.3346 | 0.563‒6.2856 |
| Anti-helicobacter pylori IgG seropositivity | Chronic sinusitis | Weighted median | 10 | 0.9788 | 0.9159 | 0.6573‒1.4575 |
| Anti-helicobacter pylori IgG seropositivity | Chronic sinusitis | Simple mode | 10 | 0.9519 | 0.868 | 0.5411‒1.6746 |
| Anti-helicobacter pylori IgG seropositivity | Chronic sinusitis | Weighted mode | 10 | 1.0152 | 0.956 | 0.6024‒1.7109 |
| Helicobacter pylori CagA antibody levels | Chronic sinusitis | Inverse variance weighted | 10 | 1.0043 | 0.8766 | 0.9516‒1.0599 |
| Helicobacter pylori CagA antibody levels | Chronic sinusitis | MR Egger | 10 | 1.0179 | 0.7819 | 0.9015‒1.1493 |
| Helicobacter pylori CagA antibody levels | Chronic sinusitis | Weighted median | 10 | 1.0339 | 0.3219 | 0.9679‒1.1045 |
| Helicobacter pylori CagA antibody levels | Chronic sinusitis | Simple mode | 10 | 1.0389 | 0.45 | 0.945‒1.1422 |
| Helicobacter pylori CagA antibody levels | Chronic sinusitis | Weighted mode | 10 | 1.0389 | 0.3957 | 0.9553‒1.1298 |
| Helicobacter pylori Catalase antibody levels | Chronic sinusitis | Inverse variance weighted | 8 | 0.9445 | 0.1427 | 0.8751‒1.0194 |
| Helicobacter pylori Catalase antibody levels | Chronic sinusitis | MR Egger | 8 | 0.8834 | 0.129 | 0.7695‒1.0142 |
| Helicobacter pylori Catalase antibody levels | Chronic sinusitis | Weighted median | 8 | 0.9129 | 0.0344 | 0.8389‒0.9934 |
| Helicobacter pylori Catalase antibody levels | Chronic sinusitis | Simple mode | 8 | 0.9231 | 0.244 | 0.8161‒1.0442 |
| Helicobacter pylori Catalase antibody levels | Chronic sinusitis | Weighted mode | 8 | 0.9008 | 0.0845 | 0.8136‒0.9974 |
| Helicobacter pylori GroEL antibody levels | Chronic sinusitis | Inverse variance weighted | 3 | 1.0968 | 0.1889 | 0.9556‒1.2588 |
| Helicobacter pylori GroEL antibody levels | Chronic sinusitis | MR Egger | 3 | 1.1117 | 0.7105 | 0.7271‒1.6996 |
| Helicobacter pylori GroEL antibody levels | Chronic sinusitis | Weighted median | 3 | 1.0787 | 0.4139 | 0.8994‒1.2937 |
| Helicobacter pylori GroEL antibody levels | Chronic sinusitis | Simple mode | 3 | 1.0578 | 0.6566 | 0.8548‒1.3091 |
| Helicobacter pylori GroEL antibody levels | Chronic sinusitis | Weighted mode | 3 | 1.0612 | 0.6335 | 0.8611‒1.3077 |
| Helicobacter pylori OMP antibody levels | Chronic sinusitis | Inverse variance weighted | 8 | 1.1937 | 0.0946 | 0.9699‒1.4692 |
| Helicobacter pylori OMP antibody levels | Chronic sinusitis | MR Egger | 8 | 0.832 | 0.6103 | 0.4254‒1.6273 |
| Helicobacter pylori OMP antibody levels | Chronic sinusitis | Weighted median | 8 | 1.0431 | 0.606 | 0.8886‒1.2245 |
| Helicobacter pylori OMP antibody levels | Chronic sinusitis | Simple mode | 8 | 0.9489 | 0.6949 | 0.7378‒1.2203 |
| Helicobacter pylori OMP antibody levels | Chronic sinusitis | Weighted mode | 8 | 1.0134 | 0.9323 | 0.7537‒1.3625 |
| Helicobacter pylori UREA antibody levels | Chronic sinusitis | Inverse variance weighted | 10 | 1.0502 | 0.1744 | 0.9785‒1.1271 |
| Helicobacter pylori UREA antibody levels | Chronic sinusitis | MR Egger | 10 | 1.1844 | 0.13 | 0.973‒1.4418 |
| Helicobacter pylori UREA antibody levels | Chronic sinusitis | Weighted median | 10 | 1.0553 | 0.259 | 0.9612‒1.1586 |
| Helicobacter pylori UREA antibody levels | Chronic sinusitis | Simple mode | 10 | 1.0548 | 0.4978 | 0.9096‒1.2233 |
| Helicobacter pylori UREA antibody levels | Chronic sinusitis | Weighted mode | 10 | 1.0573 | 0.4501 | 0.9208‒1.214 |
| Helicobacter pylori VacA antibody levels | Chronic sinusitis | Inverse variance weighted | 13 | 0.9589 | 0.3308 | 0.8811‒1.0435 |
| Helicobacter pylori VacA antibody levels | Chronic sinusitis | MR Egger | 13 | 0.8794 | 0.1502 | 0.7472‒1.0349 |
| Helicobacter pylori VacA antibody levels | Chronic sinusitis | Weighted median | 13 | 0.9963 | 0.9287 | 0.9177‒1.0816 |
| Helicobacter pylori VacA antibody levels | Chronic sinusitis | Simple mode | 13 | 1.0065 | 0.9299 | 0.8741‒1.1588 |
| Helicobacter pylori VacA antibody levels | Chronic sinusitis | Weighted mode | 13 | 0.9996 | 0.9948 | 0.8815‒1.1335 |
| Anti-helicobacter pylori IgG seropositivity | Allergic rhinitis | Inverse variance weighted | 10 | 1.0013 | 0.9409 | 0.9687‒1.0349 |
| Anti-helicobacter pylori IgG seropositivity | Allergic rhinitis | MR Egger | 10 | 1.0481 | 0.3928 | 0.9466‒1.1605 |
| Anti-helicobacter pylori IgG seropositivity | Allergic rhinitis | Weighted median | 10 | 0.9998 | 0.9929 | 0.9568‒1.0448 |
| Anti-helicobacter pylori IgG seropositivity | Allergic rhinitis | Simple mode | 10 | 1.039 | 0.3403 | 0.9644‒1.1194 |
| Anti-helicobacter pylori IgG seropositivity | Allergic rhinitis | Weighted mode | 10 | 1.0378 | 0.3475 | 0.9644‒1.1167 |
| Helicobacter pylori CagA antibody levels | Allergic rhinitis | Inverse variance weighted | 7 | 0.9987 | 0.6955 | 0.9924‒1.0051 |
| Helicobacter pylori CagA antibody levels | Allergic rhinitis | MR Egger | 7 | 1.0045 | 0.5825 | 0.9895‒1.0197 |
| Helicobacter pylori CagA antibody levels | Allergic rhinitis | Weighted median | 7 | 0.9985 | 0.7321 | 0.9898‒1.0072 |
| Helicobacter pylori CagA antibody levels | Allergic rhinitis | Simple mode | 7 | 0.9941 | 0.4304 | 0.9807‒1.0078 |
| Helicobacter pylori CagA antibody levels | Allergic rhinitis | Weighted mode | 7 | 0.9942 | 0.4383 | 0.9808‒1.0079 |
| Helicobacter pylori Catalase antibody levels | Allergic rhinitis | Inverse variance weighted | 6 | 1.0014 | 0.7734 | 0.9916‒1.0114 |
| Helicobacter pylori Catalase antibody levels | Allergic rhinitis | MR Egger | 6 | 0.9986 | 0.9079 | 0.9763‒1.0214 |
| Helicobacter pylori Catalase antibody levels | Allergic rhinitis | Weighted median | 6 | 1.0038 | 0.4857 | 0.9931‒1.0146 |
| Helicobacter pylori Catalase antibody levels | Allergic rhinitis | Simple mode | 6 | 1.0054 | 0.5045 | 0.9907‒1.0203 |
| Helicobacter pylori Catalase antibody levels | Allergic rhinitis | Weighted mode | 6 | 1.0055 | 0.4862 | 0.9911‒1.0202 |
| Helicobacter pylori GroEL antibody levels | Allergic rhinitis | Inverse variance weighted | 3 | 0.9869 | 0.0921 | 0.9718‒1.0022 |
| Helicobacter pylori GroEL antibody levels | Allergic rhinitis | MR Egger | 3 | 0.992 | 0.7386 | 0.9569‒1.0284 |
| Helicobacter pylori GroEL antibody levels | Allergic rhinitis | Weighted median | 3 | 0.992 | 0.4339 | 0.9724‒1.0121 |
| Helicobacter pylori GroEL antibody levels | Allergic rhinitis | Simple mode | 3 | 0.9924 | 0.5695 | 0.9707‒1.0146 |
| Helicobacter pylori GroEL antibody levels | Allergic rhinitis | Weighted mode | 3 | 0.9924 | 0.5857 | 0.9697‒1.0157 |
| Helicobacter pylori OMP antibody levels | Allergic rhinitis | Inverse variance weighted | 6 | 1.0031 | 0.5527 | 0.9929‒1.0134 |
| Helicobacter pylori OMP antibody levels | Allergic rhinitis | MR Egger | 6 | 1.0011 | 0.9471 | 0.97‒1.0332 |
| Helicobacter pylori OMP antibody levels | Allergic rhinitis | Weighted median | 6 | 1.0023 | 0.7264 | 0.9896‒1.0151 |
| Helicobacter pylori OMP antibody levels | Allergic rhinitis | Simple mode | 6 | 1.0024 | 0.8136 | 0.9839‒1.0212 |
| Helicobacter pylori OMP antibody levels | Allergic rhinitis | Weighted mode | 6 | 1.001 | 0.915 | 0.9839‒1.0184 |
| Helicobacter pylori UREA antibody levels | Allergic rhinitis | Inverse variance weighted | 9 | 0.9943 | 0.154 | 0.9866‒1.0021 |
| Helicobacter pylori UREA antibody levels | Allergic rhinitis | MR Egger | 9 | 0.9805 | 0.0995 | 0.9608‒1.0006 |
| Helicobacter pylori UREA antibody levels | Allergic rhinitis | Weighted median | 9 | 0.9995 | 0.9297 | 0.9889‒1.0103 |
| Helicobacter pylori UREA antibody levels | Allergic rhinitis | Simple mode | 9 | 1.0004 | 0.9657 | 0.9832‒1.0179 |
| Helicobacter pylori UREA antibody levels | Allergic rhinitis | Weighted mode | 9 | 1.0005 | 0.9522 | 0.9836‒1.0178 |
| Helicobacter pylori VacA antibody levels | Allergic rhinitis | Inverse variance weighted | 13 | 1.0009 | 0.7496 | 0.9954‒1.0065 |
| Helicobacter pylori VacA antibody levels | Allergic rhinitis | MR Egger | 13 | 0.9933 | 0.3349 | 0.9805‒1.0063 |
| Helicobacter pylori VacA antibody levels | Allergic rhinitis | Weighted median | 13 | 1.0015 | 0.7101 | 0.9938‒1.0091 |
| Helicobacter pylori VacA antibody levels | Allergic rhinitis | Simple mode | 13 | 1.0009 | 0.874 | 0.9901‒1.0118 |
| Helicobacter pylori VacA antibody levels | Allergic rhinitis | Weighted mode | 13 | 1.001 | 0.8732 | 0.9891‒1.013 |
| Anti-helicobacter pylori IgG seropositivity | Nasal polyp | Inverse variance weighted | 10 | 0.8967 | 0.65 | 0.5598‒1.4362 |
| Anti-helicobacter pylori IgG seropositivity | Nasal polyp | MR Egger | 10 | 0.5877 | 0.5511 | 0.1101‒3.1357 |
| Anti-helicobacter pylori IgG seropositivity | Nasal polyp | Weighted median | 10 | 0.821 | 0.5339 | 0.4411‒1.5283 |
| Anti-helicobacter pylori IgG seropositivity | Nasal polyp | Simple mode | 10 | 0.7515 | 0.5999 | 0.2683‒2.1049 |
| Anti-helicobacter pylori IgG seropositivity | Nasal polyp | Weighted mode | 10 | 0.7938 | 0.6793 | 0.2752‒2.2902 |
| Helicobacter pylori CagA antibody levels | Nasal polyp | Inverse variance weighted | 10 | 0.9718 | 0.6562 | 0.8566‒1.1024 |
| Helicobacter pylori CagA antibody levels | Nasal polyp | MR Egger | 10 | 1.0886 | 0.5561 | 0.8303‒1.427 |
| Helicobacter pylori CagA antibody levels | Nasal polyp | Weighted median | 10 | 1.0073 | 0.8864 | 0.9119‒1.1127 |
| Helicobacter pylori CagA antibody levels | Nasal polyp | Simple mode | 10 | 1.0061 | 0.9337 | 0.875‒1.1568 |
| Helicobacter pylori CagA antibody levels | Nasal polyp | Weighted mode | 10 | 0.9963 | 0.9473 | 0.8946‒1.1095 |
| Helicobacter pylori Catalase antibody levels | Nasal polyp | Inverse variance weighted | 8 | 0.9974 | 0.9667 | 0.8826‒1.1271 |
| Helicobacter pylori Catalase antibody levels | Nasal polyp | MR Egger | 8 | 0.8997 | 0.3886 | 0.72‒1.1243 |
| Helicobacter pylori Catalase antibody levels | Nasal polyp | Weighted median | 8 | 0.9713 | 0.6522 | 0.8557‒1.1025 |
| Helicobacter pylori Catalase antibody levels | Nasal polyp | Simple mode | 8 | 0.9224 | 0.3654 | 0.7831‒1.0864 |
| Helicobacter pylori Catalase antibody levels | Nasal polyp | Weighted mode | 8 | 0.9623 | 0.6031 | 0.8378‒1.1053 |
| Helicobacter pylori GroEL antibody levels | Nasal polyp | Inverse variance weighted | 3 | 0.998 | 0.986 | 0.8018‒1.2422 |
| Helicobacter pylori GroEL antibody levels | Nasal polyp | MR Egger | 3 | 0.8435 | 0.615 | 0.5205‒1.3667 |
| Helicobacter pylori GroEL antibody levels | Nasal polyp | Weighted median | 3 | 1.0235 | 0.8662 | 0.7808‒1.3417 |
| Helicobacter pylori GroEL antibody levels | Nasal polyp | Simple mode | 3 | 1.0707 | 0.7168 | 0.777‒1.4754 |
| Helicobacter pylori GroEL antibody levels | Nasal polyp | Weighted mode | 3 | 1.0707 | 0.7067 | 0.7864‒1.4578 |
| Helicobacter pylori OMP antibody levels | Nasal polyp | Inverse variance weighted | 8 | 1.3944 | 0.0024 | 1.1246‒1.729 |
| Helicobacter pylori OMP antibody levels | Nasal polyp | MR Egger | 8 | 1.1637 | 0.7049 | 0.5508‒2.4583 |
| Helicobacter pylori OMP antibody levels | Nasal polyp | Weighted median | 8 | 1.381 | 0.0036 | 1.111‒1.7167 |
| Helicobacter pylori OMP antibody levels | Nasal polyp | Simple mode | 8 | 1.3342 | 0.1679 | 0.9239‒1.9268 |
| Helicobacter pylori OMP antibody levels | Nasal polyp | Weighted mode | 8 | 1.3778 | 0.088 | 1.0035‒1.8918 |
| Helicobacter pylori UREA antibody levels | Nasal polyp | Inverse variance weighted | 10 | 1.0633 | 0.285 | 0.9502‒1.1899 |
| Helicobacter pylori UREA antibody levels | Nasal polyp | MR Egger | 10 | 1.3497 | 0.0973 | 0.9868‒1.8459 |
| Helicobacter pylori UREA antibody levels | Nasal polyp | Weighted median | 10 | 1.1142 | 0.1511 | 0.9613‒1.2914 |
| Helicobacter pylori UREA antibody levels | Nasal polyp | Simple mode | 10 | 1.119 | 0.3092 | 0.9121‒1.3728 |
| Helicobacter pylori UREA antibody levels | Nasal polyp | Weighted mode | 10 | 1.1321 | 0.2661 | 0.9222‒1.3898 |
| Helicobacter pylori VacA antibody levels | Nasal polyp | Inverse variance weighted | 13 | 0.8963 | 0.0849 | 0.7914‒1.0152 |
| Helicobacter pylori VacA antibody levels | Nasal polyp | MR Egger | 13 | 0.7947 | 0.0901 | 0.6237‒1.0126 |
| Helicobacter pylori VacA antibody levels | Nasal polyp | Weighted median | 13 | 0.9585 | 0.5094 | 0.8449‒1.0872 |
| Helicobacter pylori VacA antibody levels | Nasal polyp | Simple mode | 13 | 1.0079 | 0.9376 | 0.831‒1.2224 |
| Helicobacter pylori VacA antibody levels | Nasal polyp | Weighted mode | 13 | 1.0079 | 0.9253 | 0.8581‒1.1839 |
| Anti-helicobacter pylori IgG seropositivity | Acute suppurative otitis media | Inverse variance weighted | 10 | 1.1726 | 0.5855 | 0.6616‒2.0782 |
| Anti-helicobacter pylori IgG seropositivity | Acute suppurative otitis media | MR Egger | 10 | 1.5258 | 0.7091 | 0.1792‒12.993 |
| Anti-helicobacter pylori IgG seropositivity | Acute suppurative otitis media | Weighted median | 10 | 1.0554 | 0.8466 | 0.6115‒1.8215 |
| Anti-helicobacter pylori IgG seropositivity | Acute suppurative otitis media | Simple mode | 10 | 0.6355 | 0.3709 | 0.2473‒1.6325 |
| Anti-helicobacter pylori IgG seropositivity | Acute suppurative otitis media | Weighted mode | 10 | 0.7936 | 0.5693 | 0.3685‒1.7093 |
| Helicobacter pylori CagA antibody levels | Acute suppurative otitis media | Inverse variance weighted | 10 | 1.001 | 0.9724 | 0.9438‒1.0618 |
| Helicobacter pylori CagA antibody levels | Acute suppurative otitis media | MR Egger | 10 | 0.9906 | 0.8896 | 0.8711‒1.1266 |
| Helicobacter pylori CagA antibody levels | Acute suppurative otitis media | Weighted median | 10 | 1.0028 | 0.9465 | 0.9246‒1.0876 |
| Helicobacter pylori CagA antibody levels | Acute suppurative otitis media | Simple mode | 10 | 1.0003 | 0.9961 | 0.8891‒1.1254 |
| Helicobacter pylori CagA antibody levels | Acute suppurative otitis media | Weighted mode | 10 | 1.0053 | 0.9177 | 0.9125‒1.1075 |
| Helicobacter pylori Catalase antibody levels | Acute suppurative otitis media | Inverse variance weighted | 8 | 0.9343 | 0.0859 | 0.8646‒1.0096 |
| Helicobacter pylori Catalase antibody levels | Acute suppurative otitis media | MR Egger | 8 | 0.9271 | 0.3411 | 0.8032‒1.0701 |
| Helicobacter pylori Catalase antibody levels | Acute suppurative otitis media | Weighted median | 8 | 0.9417 | 0.2205 | 0.8554‒1.0367 |
| Helicobacter pylori Catalase antibody levels | Acute suppurative otitis media | Simple mode | 8 | 0.9377 | 0.358 | 0.825‒1.0659 |
| Helicobacter pylori Catalase antibody levels | Acute suppurative otitis media | Weighted mode | 8 | 0.9377 | 0.3088 | 0.836‒1.0518 |
| Helicobacter pylori GroEL antibody levels | Acute suppurative otitis media | Inverse variance weighted | 3 | 0.9043 | 0.2553 | 0.7603‒1.0754 |
| Helicobacter pylori GroEL antibody levels | Acute suppurative otitis media | MR Egger | 3 | 0.8842 | 0.6421 | 0.603‒1.2966 |
| Helicobacter pylori GroEL antibody levels | Acute suppurative otitis media | Weighted median | 3 | 0.9099 | 0.394 | 0.7323‒1.1306 |
| Helicobacter pylori GroEL antibody levels | Acute suppurative otitis media | Simple mode | 3 | 0.9151 | 0.5314 | 0.7259‒1.1537 |
| Helicobacter pylori GroEL antibody levels | Acute suppurative otitis media | Weighted mode | 3 | 0.9164 | 0.5751 | 0.708‒1.186 |
| Helicobacter pylori OMP antibody levels | Acute suppurative otitis media | Inverse variance weighted | 8 | 1.1214 | 0.299 | 0.9033‒1.3922 |
| Helicobacter pylori OMP antibody levels | Acute suppurative otitis media | MR Egger | 8 | 1.0361 | 0.9305 | 0.4823‒2.2256 |
| Helicobacter pylori OMP antibody levels | Acute suppurative otitis media | Weighted median | 8 | 1.0152 | 0.8752 | 0.8409‒1.2257 |
| Helicobacter pylori OMP antibody levels | Acute suppurative otitis media | Simple mode | 8 | 0.9913 | 0.9579 | 0.7253‒1.355 |
| Helicobacter pylori OMP antibody levels | Acute suppurative otitis media | Weighted mode | 8 | 1 | 0.9999 | 0.7441‒1.3439 |
| Helicobacter pylori UREA antibody levels | Acute suppurative otitis media | Inverse variance weighted | 10 | 0.9806 | 0.6651 | 0.8972‒1.0717 |
| Helicobacter pylori UREA antibody levels | Acute suppurative otitis media | MR Egger | 10 | 0.9979 | 0.9876 | 0.7709‒1.2917 |
| Helicobacter pylori UREA antibody levels | Acute suppurative otitis media | Weighted median | 10 | 0.9473 | 0.3672 | 0.8422‒1.0656 |
| Helicobacter pylori UREA antibody levels | Acute suppurative otitis media | Simple mode | 10 | 0.9373 | 0.4684 | 0.7927‒1.1083 |
| Helicobacter pylori UREA antibody levels | Acute suppurative otitis media | Weighted mode | 10 | 0.9406 | 0.4865 | 0.7973‒1.1097 |
| Helicobacter pylori VacA antibody levels | Acute suppurative otitis media | Inverse variance weighted | 13 | 0.9897 | 0.8323 | 0.8991‒1.0894 |
| Helicobacter pylori VacA antibody levels | Acute suppurative otitis media | MR Egger | 13 | 0.9601 | 0.691 | 0.7897‒1.1673 |
| Helicobacter pylori VacA antibody levels | Acute suppurative otitis media | Weighted median | 13 | 0.9707 | 0.519 | 0.887‒1.0624 |
| Helicobacter pylori VacA antibody levels | Acute suppurative otitis media | Simple mode | 13 | 1.0014 | 0.985 | 0.8674‒1.1561 |
| Helicobacter pylori VacA antibody levels | Acute suppurative otitis media | Weighted mode | 13 | 0.9533 | 0.408 | 0.8546‒1.0634 |
| Anti-helicobacter pylori IgG seropositivity | Chronic suppurative otitis media | Inverse variance weighted | 10 | 0.8545 | 0.6139 | 0.4639‒1.5739 |
| Anti-helicobacter pylori IgG seropositivity | Chronic suppurative otitis media | MR Egger | 10 | 0.643 | 0.6965 | 0.0757‒5.4649 |
| Anti-helicobacter pylori IgG seropositivity | Chronic suppurative otitis media | Weighted median | 10 | 0.9827 | 0.9681 | 0.4181‒2.3099 |
| Anti-helicobacter pylori IgG seropositivity | Chronic suppurative otitis media | Simple mode | 10 | 1.041 | 0.9528 | 0.2859‒3.7908 |
| Anti-helicobacter pylori IgG seropositivity | Chronic suppurative otitis media | Weighted mode | 10 | 1.1162 | 0.8339 | 0.4113‒3.0292 |
| Helicobacter pylori CagA antibody levels | Chronic suppurative otitis media | Inverse variance weighted | 14 | 0.9885 | 0.8257 | 0.8919‒1.0956 |
| Helicobacter pylori CagA antibody levels | Chronic suppurative otitis media | MR Egger | 14 | 0.7762 | 0.0499 | 0.6181‒0.9748 |
| Helicobacter pylori CagA antibody levels | Chronic suppurative otitis media | Weighted median | 14 | 1.0194 | 0.783 | 0.8893‒1.1684 |
| Helicobacter pylori CagA antibody levels | Chronic suppurative otitis media | Simple mode | 14 | 0.9295 | 0.5887 | 0.7178‒1.2036 |
| Helicobacter pylori CagA antibody levels | Chronic suppurative otitis media | Weighted mode | 14 | 1.1217 | 0.4034 | 0.8643‒1.4558 |
| Helicobacter pylori Catalase antibody levels | Chronic suppurative otitis media | Inverse variance weighted | 9 | 1.0167 | 0.8237 | 0.879‒1.176 |
| Helicobacter pylori Catalase antibody levels | Chronic suppurative otitis media | MR Egger | 9 | 0.9632 | 0.83 | 0.6927‒1.3393 |
| Helicobacter pylori Catalase antibody levels | Chronic suppurative otitis media | Weighted median | 9 | 1.0348 | 0.7316 | 0.8513‒1.2578 |
| Helicobacter pylori Catalase antibody levels | Chronic suppurative otitis media | Simple mode | 9 | 1.0768 | 0.6465 | 0.7942‒1.46 |
| Helicobacter pylori Catalase antibody levels | Chronic suppurative otitis media | Weighted mode | 9 | 1.0652 | 0.6383 | 0.8267‒1.3724 |
| Helicobacter pylori GroEL antibody levels | Chronic suppurative otitis media | Inverse variance weighted | 4 | 1.1272 | 0.3403 | 0.8812‒1.4419 |
| Helicobacter pylori GroEL antibody levels | Chronic suppurative otitis media | MR Egger | 4 | 1.0901 | 0.866 | 0.4502‒2.6395 |
| Helicobacter pylori GroEL antibody levels | Chronic suppurative otitis media | Weighted median | 4 | 1.0146 | 0.9264 | 0.7455‒1.3809 |
| Helicobacter pylori GroEL antibody levels | Chronic suppurative otitis media | Simple mode | 4 | 0.9996 | 0.9986 | 0.6402‒1.5606 |
| Helicobacter pylori GroEL antibody levels | Chronic suppurative otitis media | Weighted mode | 4 | 1.0046 | 0.9809 | 0.7121‒1.4172 |
| Helicobacter pylori OMP antibody levels | Chronic suppurative otitis media | Inverse variance weighted | 9 | 0.9092 | 0.2341 | 0.7773‒1.0635 |
| Helicobacter pylori OMP antibody levels | Chronic suppurative otitis media | MR Egger | 9 | 0.8794 | 0.6404 | 0.5247‒1.4737 |
| Helicobacter pylori OMP antibody levels | Chronic suppurative otitis media | Weighted median | 9 | 0.9379 | 0.4721 | 0.7874‒1.1171 |
| Helicobacter pylori OMP antibody levels | Chronic suppurative otitis media | Simple mode | 9 | 0.9544 | 0.7299 | 0.7392‒1.2324 |
| Helicobacter pylori OMP antibody levels | Chronic suppurative otitis media | Weighted mode | 9 | 0.8957 | 0.356 | 0.7186‒1.1165 |
| Helicobacter pylori UREA antibody levels | Chronic suppurative otitis media | Inverse variance weighted | 10 | 0.989 | 0.8453 | 0.8852‒1.105 |
| Helicobacter pylori UREA antibody levels | Chronic suppurative otitis media | MR Egger | 10 | 1.2876 | 0.1996 | 0.9034‒1.8352 |
| Helicobacter pylori UREA antibody levels | Chronic suppurative otitis media | Weighted median | 10 | 0.9669 | 0.6414 | 0.8392‒1.114 |
| Helicobacter pylori UREA antibody levels | Chronic suppurative otitis media | Simple mode | 10 | 0.9123 | 0.3567 | 0.7582‒1.0978 |
| Helicobacter pylori UREA antibody levels | Chronic suppurative otitis media | Weighted mode | 10 | 0.9544 | 0.5603 | 0.8205‒1.1102 |
| Helicobacter pylori VacA antibody levels | Chronic suppurative otitis media | Inverse variance weighted | 15 | 0.9903 | 0.8251 | 0.9084‒1.0796 |
| Helicobacter pylori VacA antibody levels | Chronic suppurative otitis media | MR Egger | 15 | 1.0089 | 0.9182 | 0.8554‒1.1898 |
| Helicobacter pylori VacA antibody levels | Chronic suppurative otitis media | Weighted median | 15 | 0.9942 | 0.9228 | 0.8831‒1.1192 |
| Helicobacter pylori VacA antibody levels | Chronic suppurative otitis media | Simple mode | 15 | 0.915 | 0.3335 | 0.769‒1.0887 |
| Helicobacter pylori VacA antibody levels | Chronic suppurative otitis media | Weighted mode | 15 | 0.9979 | 0.9736 | 0.8847‒1.1256 |
| Anti-helicobacter pylori IgG seropositivity | Nonsuppurative otitis media | Inverse variance weighted | 10 | 0.8742 | 0.5125 | 0.5847‒1.3071 |
| Anti-helicobacter pylori IgG seropositivity | Nonsuppurative otitis media | MR Egger | 10 | 0.4911 | 0.3574 | 0.1178‒2.0467 |
| Anti-helicobacter pylori IgG seropositivity | Nonsuppurative otitis media | Weighted median | 10 | 1.0745 | 0.7856 | 0.6404‒1.8028 |
| Anti-helicobacter pylori IgG seropositivity | Nonsuppurative otitis media | Simple mode | 10 | 1.2102 | 0.6626 | 0.5283‒2.7724 |
| Anti-helicobacter pylori IgG seropositivity | Nonsuppurative otitis media | Weighted mode | 10 | 1.1634 | 0.7097 | 0.5376‒2.5178 |
| Helicobacter pylori CagA antibody levels | Nonsuppurative otitis media | Inverse variance weighted | 10 | 1.0778 | 0.021 | 1.0114‒1.1487 |
| Helicobacter pylori CagA antibody levels | Nonsuppurative otitis media | MR Egger | 10 | 1.0756 | 0.3232 | 0.9391‒1.232 |
| Helicobacter pylori CagA antibody levels | Nonsuppurative otitis media | Weighted median | 10 | 1.072 | 0.14 | 0.9775‒1.1756 |
| Helicobacter pylori CagA antibody levels | Nonsuppurative otitis media | Simple mode | 10 | 1.0768 | 0.3375 | 0.9331‒1.2426 |
| Helicobacter pylori CagA antibody levels | Nonsuppurative otitis media | Weighted mode | 10 | 1.0566 | 0.368 | 0.9429‒1.1839 |
| Helicobacter pylori Catalase antibody levels | Nonsuppurative otitis media | Inverse variance weighted | 8 | 0.9602 | 0.3439 | 0.8827‒1.0445 |
| Helicobacter pylori Catalase antibody levels | Nonsuppurative otitis media | MR Egger | 8 | 0.9407 | 0.4713 | 0.8049‒1.0994 |
| Helicobacter pylori Catalase antibody levels | Nonsuppurative otitis media | Weighted median | 8 | 0.9474 | 0.3285 | 0.85‒1.0559 |
| Helicobacter pylori Catalase antibody levels | Nonsuppurative otitis media | Simple mode | 8 | 0.9583 | 0.5697 | 0.8333‒1.1022 |
| Helicobacter pylori Catalase antibody levels | Nonsuppurative otitis media | Weighted mode | 8 | 0.9476 | 0.4031 | 0.8417‒1.0669 |
| Helicobacter pylori GroEL antibody levels | Nonsuppurative otitis media | Inverse variance weighted | 3 | 0.9828 | 0.8563 | 0.8144‒1.1859 |
| Helicobacter pylori GroEL antibody levels | Nonsuppurative otitis media | MR Egger | 3 | 1.0468 | 0.8643 | 0.6916‒1.5846 |
| Helicobacter pylori GroEL antibody levels | Nonsuppurative otitis media | Weighted median | 3 | 1.0046 | 0.9687 | 0.7981‒1.2645 |
| Helicobacter pylori GroEL antibody levels | Nonsuppurative otitis media | Simple mode | 3 | 1.0309 | 0.8446 | 0.7883‒1.3482 |
| Helicobacter pylori GroEL antibody levels | Nonsuppurative otitis media | Weighted mode | 3 | 1.0246 | 0.8703 | 0.7921‒1.3254 |
| Helicobacter pylori OMP antibody levels | Nonsuppurative otitis media | Inverse variance weighted | 8 | 1.0884 | 0.4187 | 0.8864‒1.3364 |
| Helicobacter pylori OMP antibody levels | Nonsuppurative otitis media | MR Egger | 8 | 0.8752 | 0.7249 | 0.431‒1.7774 |
| Helicobacter pylori OMP antibody levels | Nonsuppurative otitis media | Weighted median | 8 | 1.1243 | 0.2622 | 0.9161‒1.3798 |
| Helicobacter pylori OMP antibody levels | Nonsuppurative otitis media | Simple mode | 8 | 0.7861 | 0.2952 | 0.5182‒1.1927 |
| Helicobacter pylori OMP antibody levels | Nonsuppurative otitis media | Weighted mode | 8 | 1.3687 | 0.1104 | 0.9774‒1.9166 |
| Helicobacter pylori UREA antibody levels | Nonsuppurative otitis media | Inverse variance weighted | 10 | 1.0909 | 0.0754 | 0.9911‒1.2008 |
| Helicobacter pylori UREA antibody levels | Nonsuppurative otitis media | MR Egger | 10 | 1.0829 | 0.5747 | 0.8293‒1.414 |
| Helicobacter pylori UREA antibody levels | Nonsuppurative otitis media | Weighted median | 10 | 1.0517 | 0.4201 | 0.9305‒1.1886 |
| Helicobacter pylori UREA antibody levels | Nonsuppurative otitis media | Simple mode | 10 | 1.0143 | 0.8872 | 0.8378‒1.2281 |
| Helicobacter pylori UREA antibody levels | Nonsuppurative otitis media | Weighted mode | 10 | 1.0228 | 0.8222 | 0.8448‒1.2384 |
| Helicobacter pylori VacA antibody levels | Nonsuppurative otitis media | Inverse variance weighted | 13 | 0.9886 | 0.8123 | 0.8989‒1.0871 |
| Helicobacter pylori VacA antibody levels | Nonsuppurative otitis media | MR Egger | 13 | 0.9621 | 0.7034 | 0.7928‒1.1676 |
| Helicobacter pylori VacA antibody levels | Nonsuppurative otitis media | Weighted median | 13 | 0.9505 | 0.3183 | 0.8602‒1.0502 |
| Helicobacter pylori VacA antibody levels | Nonsuppurative otitis media | Simple mode | 13 | 0.9295 | 0.3193 | 0.8098‒1.0669 |
| Helicobacter pylori VacA antibody levels | Nonsuppurative otitis media | Weighted mode | 13 | 0.9367 | 0.2429 | 0.8439‒1.0397 |
| Anti-helicobacter pylori IgG seropositivity | Sleep apnoea | Wald ratio | 1 | 0.9934 | 0.1488 | 0.9845‒1.0024 |
| Helicobacter pylori CagA antibody levels | Sleep apnoea | Inverse variance weighted | 2 | 1.0001 | 0.8285 | 0.9991‒1.0011 |
| Helicobacter pylori Catalase antibody levels | Sleep apnoea | Inverse variance weighted | 2 | 1.0006 | 0.3 | 0.9994‒1.0019 |
| Helicobacter pylori GroEL antibody levels | Sleep apnoea | Inverse variance weighted | 2 | 0.9994 | 0.4353 | 0.9978‒1.001 |
| Helicobacter pylori OMP antibody levels | Sleep apnoea | Inverse variance weighted | 2 | 0.9989 | 0.1214 | 0.9975‒1.0003 |
| Helicobacter pylori UREA antibody levels | Sleep apnoea | Inverse variance weighted | 4 | 1.0002 | 0.7343 | 0.9992‒1.0012 |
| Helicobacter pylori UREA antibody levels | Sleep apnoea | MR Egger | 4 | 1.0014 | 0.732 | 0.9946‒1.0081 |
| Helicobacter pylori UREA antibody levels | Sleep apnoea | Weighted median | 4 | 1.0003 | 0.6573 | 0.9991‒1.0015 |
| Helicobacter pylori UREA antibody levels | Sleep apnoea | Simple mode | 4 | 1.0007 | 0.4864 | 0.999‒1.0023 |
| Helicobacter pylori UREA antibody levels | Sleep apnoea | Weighted mode | 4 | 1.0006 | 0.5055 | 0.999‒1.0022 |
| Helicobacter pylori VacA antibody levels | Sleep apnoea | Inverse variance weighted | 2 | 0.9994 | 0.3143 | 0.9982‒1.0006 |
| Anti-helicobacter pylori IgG seropositivity | Laryngeal cancer | Inverse variance weighted | 7 | 0.9999 | 0.8638 | 0.9985‒1.0012 |
| Anti-helicobacter pylori IgG seropositivity | Laryngeal cancer | MR Egger | 7 | 1.0016 | 0.7071 | 0.9939‒1.0092 |
| Anti-helicobacter pylori IgG seropositivity | Laryngeal cancer | Weighted median | 7 | 1.0003 | 0.7121 | 0.9985‒1.0022 |
| Anti-helicobacter pylori IgG seropositivity | Laryngeal cancer | Simple mode | 7 | 1.0004 | 0.815 | 0.9975‒1.0032 |
| Anti-helicobacter pylori IgG seropositivity | Laryngeal cancer | Weighted mode | 7 | 1.0004 | 0.8097 | 0.9976‒1.0031 |
| Helicobacter pylori CagA antibody levels | Laryngeal cancer | Inverse variance weighted | 9 | 1 | 0.8325 | 0.9998‒1.0002 |
| Helicobacter pylori CagA antibody levels | Laryngeal cancer | MR Egger | 9 | 1.0001 | 0.7922 | 0.9996‒1.0006 |
| Helicobacter pylori CagA antibody levels | Laryngeal cancer | Weighted median | 9 | 1 | 0.7963 | 0.9998‒1.0003 |
| Helicobacter pylori CagA antibody levels | Laryngeal cancer | Simple mode | 9 | 1 | 0.8226 | 0.9997‒1.0004 |
| Helicobacter pylori CagA antibody levels | Laryngeal cancer | Weighted mode | 9 | 1 | 0.8638 | 0.9997‒1.0004 |
| Helicobacter pylori Catalase antibody levels | Laryngeal cancer | Inverse variance weighted | 3 | 0.9997 | 0.1133 | 0.9992‒1.0001 |
| Helicobacter pylori Catalase antibody levels | Laryngeal cancer | MR Egger | 3 | 0.9983 | 0.7642 | 0.9899‒1.0069 |
| Helicobacter pylori Catalase antibody levels | Laryngeal cancer | Weighted median | 3 | 0.9995 | 0.0823 | 0.999‒1.0001 |
| Helicobacter pylori Catalase antibody levels | Laryngeal cancer | Simple mode | 3 | 0.9994 | 0.2553 | 0.9988‒1.0001 |
| Helicobacter pylori Catalase antibody levels | Laryngeal cancer | Weighted mode | 3 | 0.9994 | 0.2585 | 0.9988‒1.0001 |
| Helicobacter pylori GroEL antibody levels | Laryngeal cancer | Inverse variance weighted | 2 | 1.0003 | 0.3912 | 0.9996‒1.001 |
| Helicobacter pylori OMP antibody levels | Laryngeal cancer | Inverse variance weighted | 5 | 0.9998 | 0.2189 | 0.9994‒1.0001 |
| Helicobacter pylori OMP antibody levels | Laryngeal cancer | MR Egger | 5 | 0.9997 | 0.7716 | 0.9979‒1.0015 |
| Helicobacter pylori OMP antibody levels | Laryngeal cancer | Weighted median | 5 | 0.9999 | 0.663 | 0.9994‒1.0004 |
| Helicobacter pylori OMP antibody levels | Laryngeal cancer | Simple mode | 5 | 0.9999 | 0.7374 | 0.9993‒1.0005 |
| Helicobacter pylori OMP antibody levels | Laryngeal cancer | Weighted mode | 5 | 0.9999 | 0.6772 | 0.9992‒1.0005 |
| Helicobacter pylori UREA antibody levels | Laryngeal cancer | Inverse variance weighted | 7 | 1 | 0.8739 | 0.9997‒1.0004 |
| Helicobacter pylori UREA antibody levels | Laryngeal cancer | MR Egger | 7 | 0.9992 | 0.1679 | 0.9982‒1.0002 |
| Helicobacter pylori UREA antibody levels | Laryngeal cancer | Weighted median | 7 | 1.0001 | 0.5346 | 0.9997‒1.0006 |
| Helicobacter pylori UREA antibody levels | Laryngeal cancer | Simple mode | 7 | 1.0004 | 0.3722 | 0.9996‒1.0011 |
| Helicobacter pylori UREA antibody levels | Laryngeal cancer | Weighted mode | 7 | 1.0003 | 0.4159 | 0.9996‒1.0011 |
| Helicobacter pylori VacA antibody levels | Laryngeal cancer | Inverse variance weighted | 8 | 0.9999 | 0.6069 | 0.9997‒1.0002 |
| Helicobacter pylori VacA antibody levels | Laryngeal cancer | MR Egger | 8 | 1.0005 | 0.2982 | 0.9997‒1.0013 |
| Helicobacter pylori VacA antibody levels | Laryngeal cancer | Weighted median | 8 | 1 | 0.8354 | 0.9996‒1.0003 |
| Helicobacter pylori VacA antibody levels | Laryngeal cancer | Simple mode | 8 | 1 | 0.9553 | 0.9995‒1.0006 |
| Helicobacter pylori VacA antibody levels | Laryngeal cancer | Weighted mode | 8 | 1 | 0.9238 | 0.9995‒1.0005 |

**Supplementary Table 4** Sensitivity analyses of the MR analysis results of exposure and outcomes.

| **Exposure** | **Outcomes** | **Heterogeneity** | | | | | | **Horizontal pleiotropy** | | | **MR-PRESSO** | |
| --- | --- | --- | --- | --- | --- | --- | --- | --- | --- | --- | --- | --- |
|  |  | **MR-Egger** | | | **IVW** | | | **MR-Egger** | | | **Global Test** | |
|  |  | **Q** | **Q-df** | **p** | **Q** | **Q-df** | **p** | **Egger intercept** | **Se** | **p** | **RSSobs** | **p** |
| Helicobacter pylori CagA antibody levels | Nonsuppurative otitis media | 6,9884 | 8 | 0.5379 | 6,9895 | 9 | 0.6382 | 0.0009 | 0.0274 | 0.974 | 9,1868 | 0.617 |

**Supplementary Figure 1** Leave-one-out analysis for H. pylori CagA antibody levels on nonsuppurative otitis media.


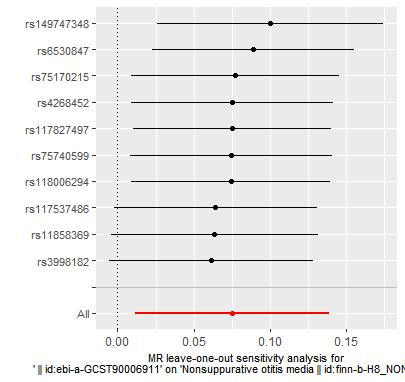


**Supplementary Figure 2** Funnel plot for H. pylori CagA antibody levels on nonsuppurative otitis media.


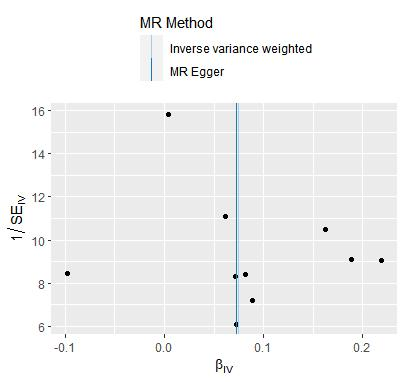


**Supplementary Figure 3** Scatter plot for H. pylori CagA antibody levels on nonsuppurative otitis media.


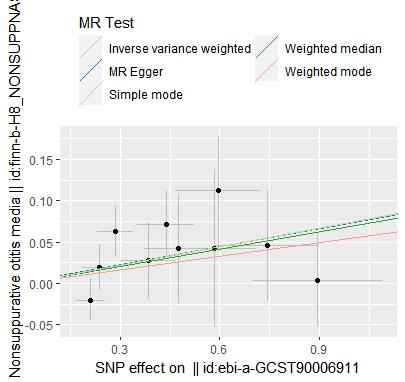


**Supplementary Figure 4** MR effect size for H. pylori CagA antibody levels on nonsuppurative otitis media.


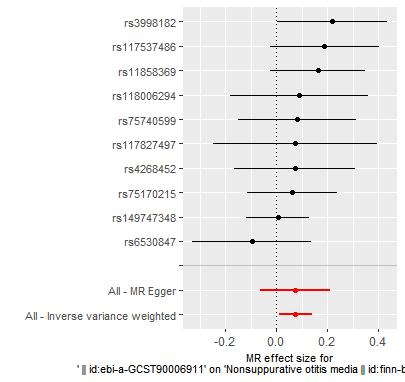

Supplement: Supplementary file 1 [file mmc1.docx]
